# A Mur Regulator Protein in the Extremophilic Bacterium *Deinococcus radiodurans*

**DOI:** 10.1371/journal.pone.0106341

**Published:** 2014-09-22

**Authors:** Amir Miraj Ul Hussain Shah, Ye Zhao, Yunfei Wang, Guoquan Yan, Qikun Zhang, Liangyan Wang, Bing Tian, Huan Chen, Yuejin Hua

**Affiliations:** 1 Key Laboratory of Chinese Ministry of Agriculture for Nuclear-Agricultural Sciences, Institute of Nuclear-Agricultural Sciences, Zhejiang University, Hangzhou, China; 2 Key Laboratory of Laboratory Medicine, Ministry of Education, Zhejiang Provincial Key Laboratory of Medical Genetics, School of Laboratory Medicine & Life Science, Wenzhou Medical College, Wenzhou, China; 3 Laboratory of Microbiology and Genomics, Zhejiang Institute of Microbiology, Hangzhou, China; Baylor College of Medicine, United States of America

## Abstract

Ferric uptake regulator (Fur) is a transcriptional regulator that controls the expression of genes involved in the uptake of iron and manganese, as well as vital nutrients, and is essential for intracellular redox cycling. We identified a unique Fur homolog (DR0865) from *Deinococcus radiodurans*, which is known for its extreme resistance to radiation and oxidants. A *dr0865* mutant (Mt-0865) showed a higher sensitivity to manganese stress, hydrogen peroxide, gamma irradiation and ultraviolet (UV) irradiation than the wild-type R1 strain. Cellular manganese (Mn) ion (Mn^2+^) analysis showed that Mn^2+^, copper (Cu^2+^), and ferric (Fe^3+^) ions accumulated significantly in the mutant, which suggests that the *dr0865* gene is not only involved in the regulation of Mn^2+^ homeostasis, but also affects the uptake of other ions. In addition, transcriptome profiles under MnCl_2_ stress showed that the expression of many genes involved in Mn metabolism was significantly different in the wild-type R1 and DR0865 mutant (Mt-0865). Furthermore, we found that the *dr0865* gene serves as a positive regulator of the manganese efflux pump gene *mntE* (*dr1236*), and as a negative regulator of Mn ABC transporter genes, such as *dr2283*, *dr2284* and *dr2523*. Therefore, it plays an important role in maintaining the homoeostasis of intracellular Mn (II), and also other Mn^2+^, zinc (Zn^2+^) and Cu^2+^ ions. Based on its role in manganese homeostasis, DR0865 likely belongs to the Mur sub-family of Fur homolog.

## Introduction

Metal ions, such as manganese (Mn^2+^) and iron (Fe^2+^), are essential micronutrients for many microorganisms and act as enzyme cofactors for a wide range of proteins in processes such as DNA synthesis, DNA repair, reactive oxygen species (ROS) scavenging and electron transport [1]. However, when in excess, they are toxic to cells. Excess iron induces the over-production of harmful ROS, such as super-oxide anion radicals (O_2_^−^) and hydrogen peroxide (H_2_O_2_) [1]. High levels of ROS may target DNA, RNA, proteins and lipids through the hydroxyl radicals (HO•) that are generated from H_2_O_2_ in the Fenton reaction, which uses divalent ions [2]. Inhibition of RNA and protein synthesis occur when high intracellular levels of manganese are reached [3]. Therefore, microorganisms have evolved efficient mechanisms to maintain metal ion homeostasis [4].

The uptake of metal ions is controlled by the ferric uptake regulator (Fur) or the *Diphtheria* toxin repressor (DtxR) family of proteins [5]. The Fur superfamily comprises different proteins with distinct regulatory roles [6]. Fur and Zur (zinc uptake regulator) [7], which respond to iron (Fe^2+^) or zinc (Zn^2+^), respectively, repress, the expression of genes involved in Fe^2+^ or Zn^2+^ uptake. The PerR protein, which has been found in Gram-positive bacteria such as *Bacillus* and *Staphylococcus*, regulates several genes that are involved in the oxidative stress response [8], [9]. Another Fur homolog, named Irr, which can repress the heme biosynthesis pathway, was first found in *Bradyrhizobium japonicum* [10], [11]. In 2004, Johnston’s group identified a new *fur*-like protein named Mur (manganese uptake regulator) in *Rhizobium leguminosarum*. This protein represses the transcription of the *sitABCD* genes in response to Mn^2+^ [12].

*Deinococcus radiodurans* is a well-known bacterium that has extraordinary resistance to ionizing radiation (IR), ultraviolet radiation (UV), various DNA-damaging agents, oxidative stress and desiccation [13]. Ionizing radiation can directly damage bio-macromolecules and also produces ROS, which can attack both proteins and DNA [14]. Recently, it has been shown that *D. radiodurans* has a special Mn/Fe regulatory system, which accumulates exceptionally high levels of intracellular Mn^2+^ and low levels of Fe^2+^. Mn^2+^ may act as an antioxidant to strengthen or support the antioxidant enzyme system, which protects the bacteria from oxidative stress [15], [16]. It was shown that there are three types of Mn^2+^-dependent transport genes in *D. radiodurans*: *dr1236* (Mn^2+^ efflux genes) [17], *dr1709* (Nramp family transporters) and three ATP-dependent transporters (*dr2283*, *dr2284* and *dr2523*). The genes that are involved in Fe^2+^-dependent transport encode an ABC-type hemin transporter (*drb0016*), an ABC-type Fe(III)-siderophore transporter (*drb0017*), two Fe(II) transporters (*dr1219*, *dr1220*) and two DNA protection proteins (Dps) (*dr2263*, *drb0092*) [1]. Furthermore, *D. radiodurans* also has three oxidation-related regulators: OxyR (DR0615), DtxR (DR2539), and a Fur homolog (DR0865) [1]. The DR0615 protein is both a transcriptional activator of the *katE* and *drb0125* genes and a transcriptional repressor of the *dps* and *mntH* genes [18]. The DR2539 protein acts as a negative regulator of a Mn^2+^ transporter gene (*dr2283*) and as a positive regulator of Fe^2+^-dependent transporter genes (*dr1219* and *drb0125*) [19]. However, the function of the Fur homolog (DR0865) is still unknown.

In this study, we aimed to elucidate the function of the Fur homolog (DR0865) and demonstrate its role in maintaining the homoeostasis of intracellular Mn. The results showed that DR0865 is not only a Mur protein, but is also vital for the homoeostasis of intracellular Mn^2+^, Zn^2+^ and Cu^2+^ ions.

## Results

### *D. radiodurans* gene encodes a putative Fur family protein

**Figure 1:**
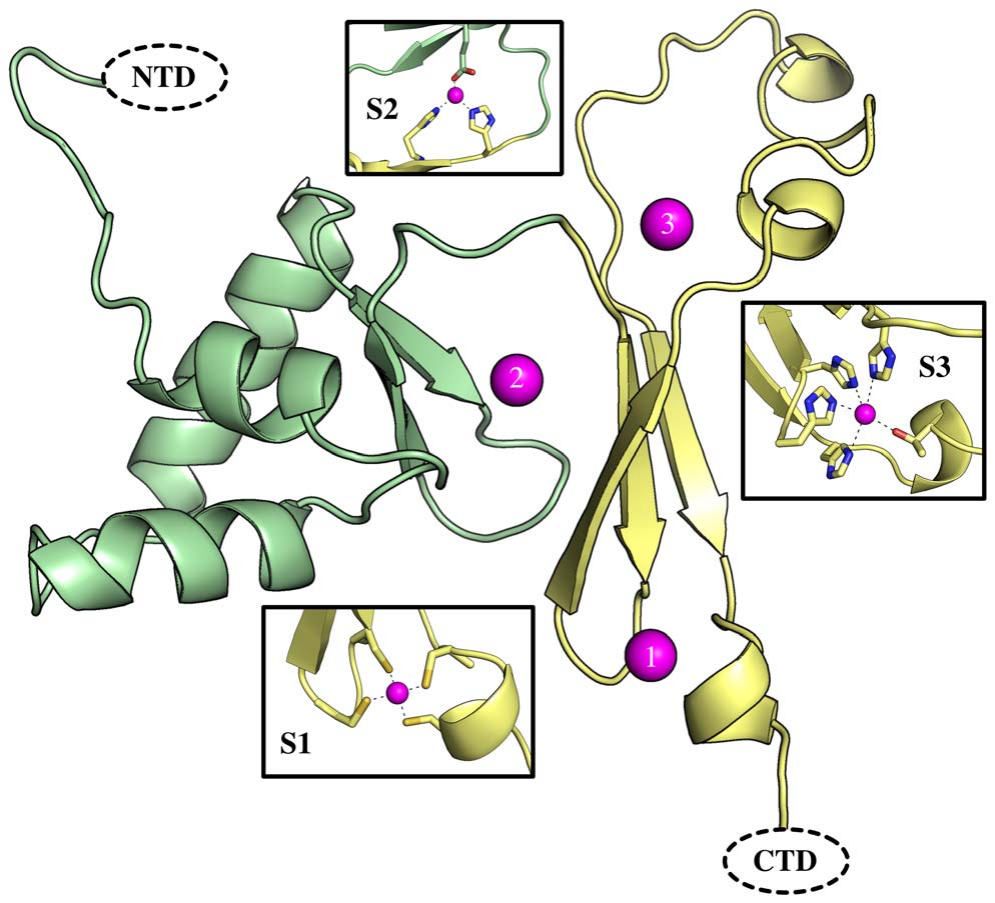
The predicted structure of DR0865 was obtained from homology modeling (Swiss-model) using Hpy-Fur PDB: 2XIG as starting model.

There is a potential *fur* homolog (*dr0865*), which encodes a protein that contains 132 amino acids, in *D. radiodurans* genome. A BLASTP analysis showed that DR0865 exhibits 24% identity to *Helicobacter pylori* Fur (Hpy-Fur) and 26% identity to the *E. coli* Fur protein. Further comparison with the Hpy-Fur sequence showed that DR0865 has three similar metal-binding domains. Domain I consists of amino acid residues C82, C85, C121 and C124, domain II comprises the residues E70, H77 and H79 and domain III is formed from residues H76, H92, T97, H113 and H78 (Figure S1 in File S1). The predicted structure of DR0865 is based on the crystal structure of Hpy-Fur (Figure 1). Previous data showed that ZnS_4_ binding by domain I stabilizes β3-β4-β5 structures. Domain II is a metal sensing site, which can regulate DNA binding ability in response to changes in metal concentrations. Domain III is not necessary for DNA binding, however, mutation of this domain reduces the DNA binding ability [20].

### The absence of *dr0865* inhibits cell growth

**Figure 2:**
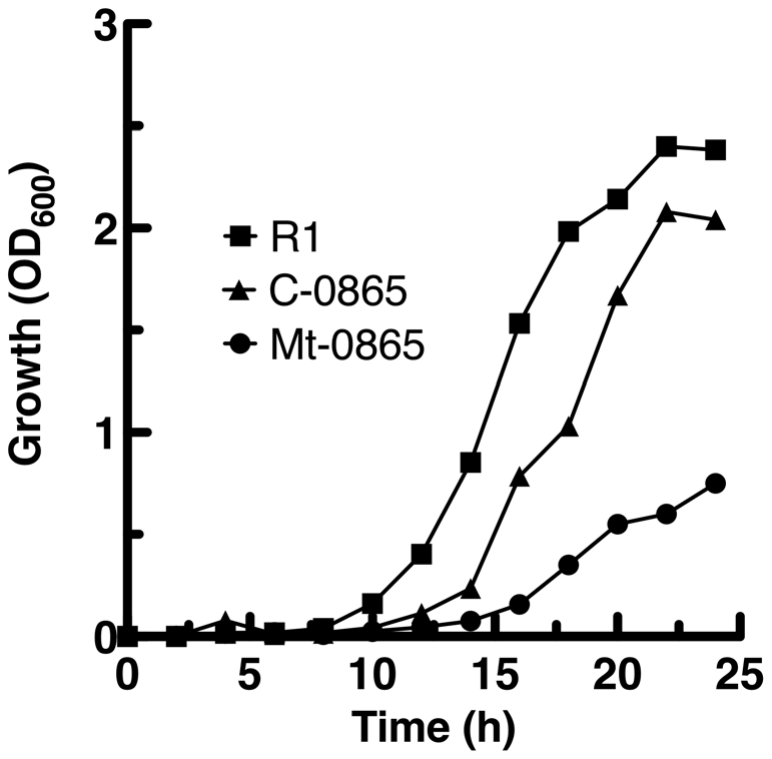
Growth curves of the wild-type R1 strain (black square), complement C-0865 strain (black triangle) and Mt-0865 strain (black circle). Data represent the mean ± standard deviation of three independent experiments.

To confirm the specific roles of DR0865 in *D. radiodurans*, the null mutant of *dr0865* (Mt-0865) and the complemented strain (C-0865) were constructed. The coding region of the *dr0865* gene was replaced with a kanamycin resistance cassette under a constitutively expressed *D. radiodurans groEL* promoter (Figure S2 in File S1). As shown in Figure 2, the cell growth of Mt-0865 was approximately two-fold lower than that of the wild type strain at 30°C, whereas the growth rate of the complemented strain C-0865 was similar to that of the wild-type strain. This result indicates that the *dr0865* gene is necessary for cell growth and other metabolic activities.

### Loss of *dr0865* causes Mn (II) ion sensitivity in *D. radiodurans*

**Figure 3:**
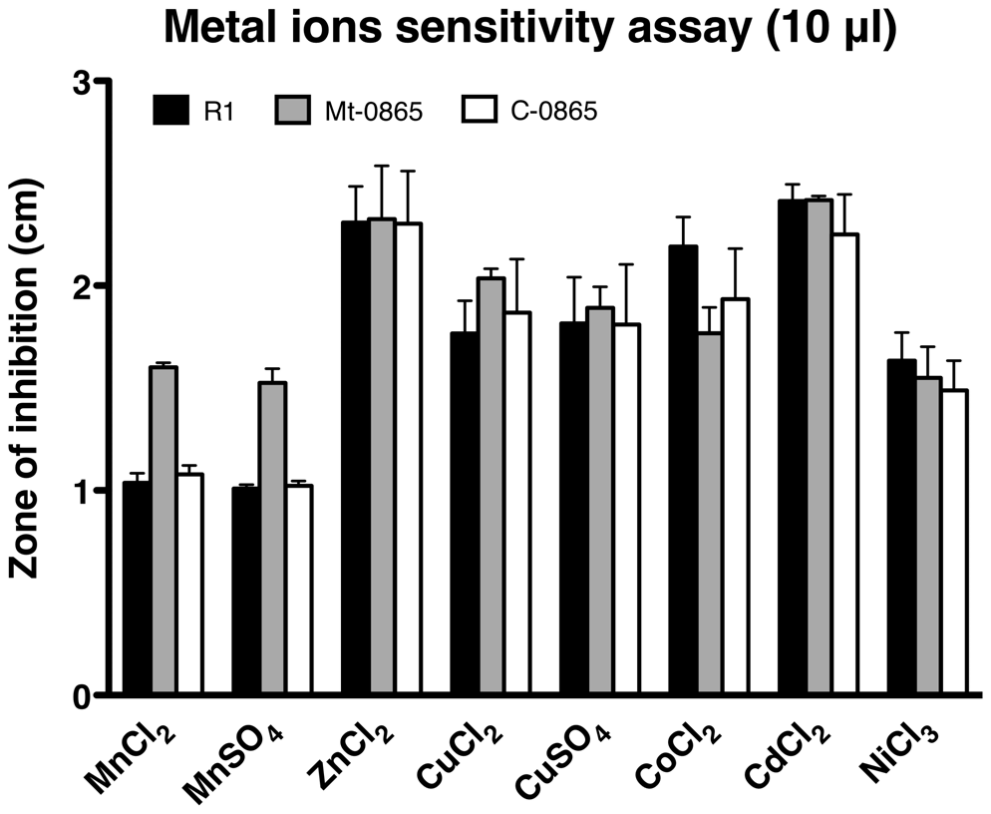
The zone of inhibition of the wild-type R1 (black bar), Mt-0865 (gray bar) and C-0865 strains (white bar) respectively, under various cations stress. Strains were cultured in TGY plates, overlaid with filter discs saturated with 1 M solution of various cations. The zone of inhibition was measured from the edge of the disc after 3 days. Data represent the means ± standard deviation of three independent experiments.

To test whether the growth inhibition was caused by a disruption of ion homeostasis, a metal ion sensitivity assay was carried out as described previously [17]. As shown in Figure 3 and Figure S3 in File S1, the growth of Mt-0865 was strongly inhibited by Mn^2+^ but not by the presence of other metal ions. The C-0865 showed the same growth phenotype as the wild-type R1 strain, which indicates that mutation of the *dr0865* gene disrupts Mn^2+^ homeostasis.

**Figure 4:**
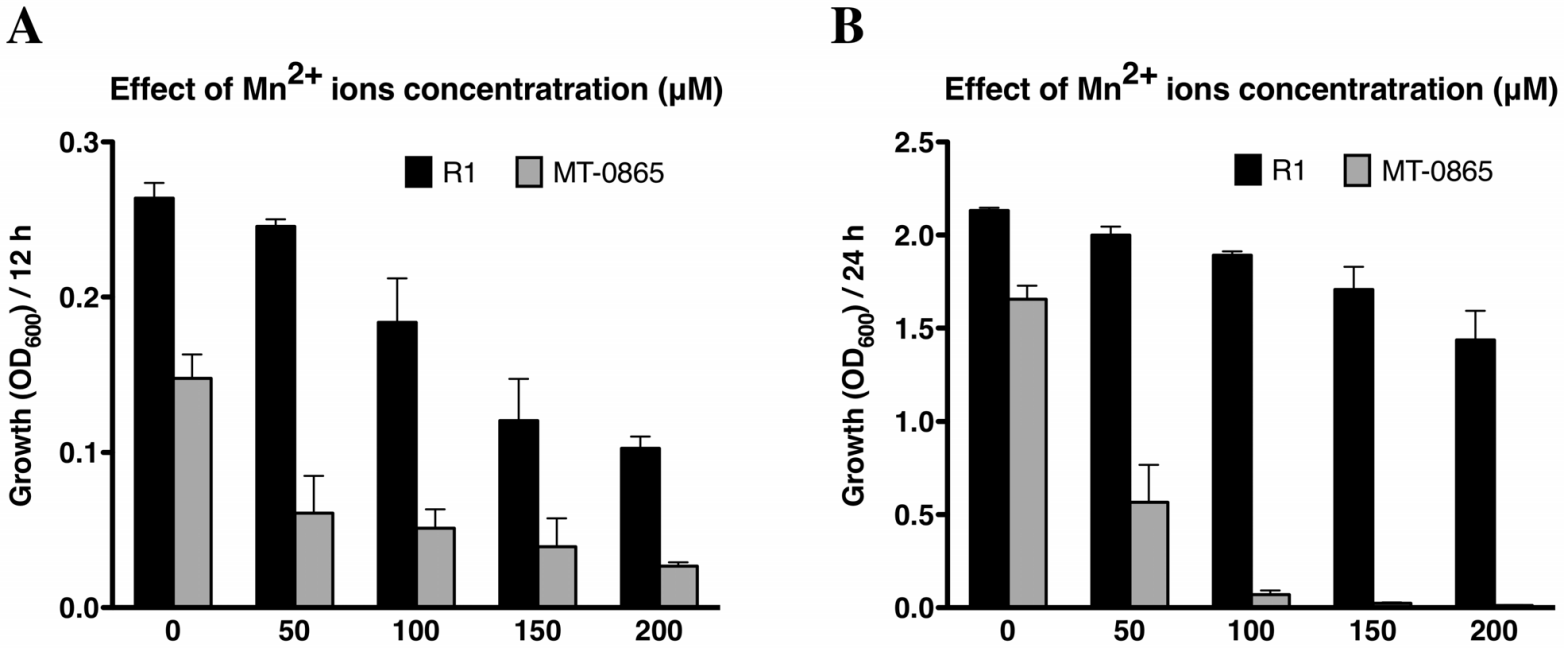
Sensitivity of the wild-type R1 strain (black bar) and mutant Mt-0865 strain (gray bar) to MnCl_2_. Strains were cultured in TGY, supplemented with 0, 50,100 or 150 µM of MnCl_2_. The OD_600_ was measured after 12 and 24 h. Data represent the means ± standard deviation of three independent experiments.

To further confirm the Mn sensitivity of Mt-0865, we measured the effect of various concentrations of Mn^2+^ on the growth of Mt-0865 (Figure 4). In comparison with the wild-type R1 strain, the growth of Mt-0865 was inhibited in the presence of low concentrations of Mn^2+^ in TGY medium. When the Mn^2+^ concentration was increased, the growth defect phenotype became more pronounced. An analogous was observed in previous studies, in which the growth of a *Streptococcus pneumoniae* mutant with a disrupted calcium efflux system was more severely inhibited at higher calcium concentrations [21]. Therefore, we inferred that the Mt-0865 strain may either have a higher rate of Mn^2+^ uptake or is unable to efficiently remove excess Mn^2+^.

### The effect of H_2_O_2_, UV and gamma irradiation on the survival of Mt-0865

**Figure 5:**
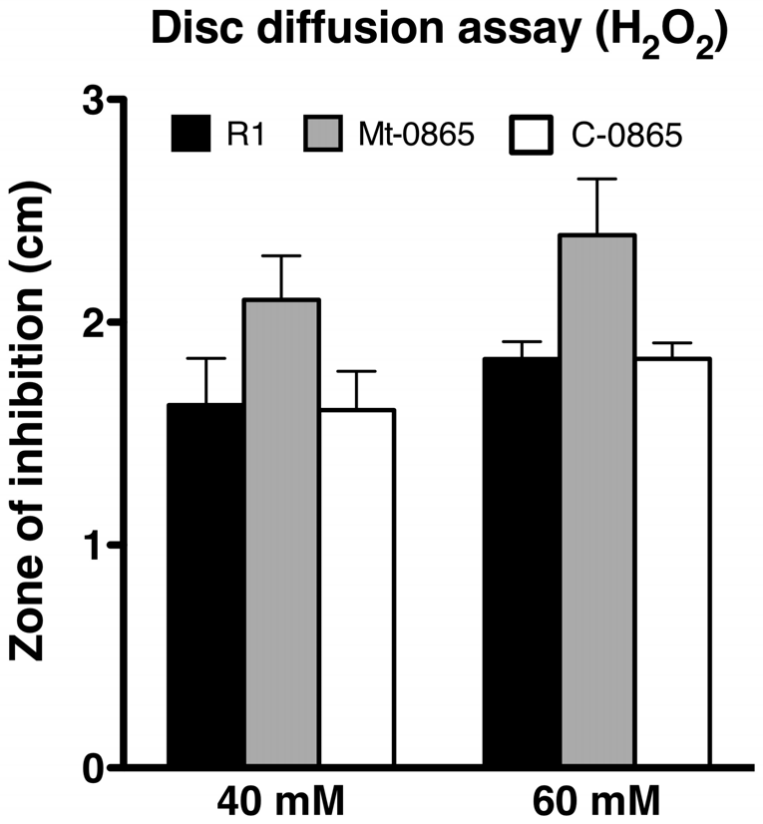
Hydrogen peroxide sensitivity assay for the wild-type R1, Mt-0865 and C-0865 strains, The wild-type R1 strain (black bar), Mt-0865 strain (gray bar) and C-0865 strain (white bar) were cultured in TGY plates, overlaid with filter discs saturated with 4 µl and 6 µl of 1 M solution of H_2_O_2_.

**Figure 6:**
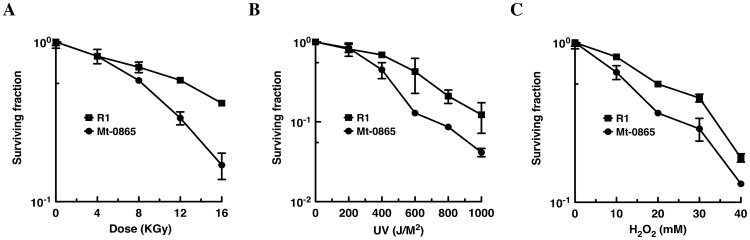
Survival curves of *D. radiodurans* strains exposed to (A) ionizing irradiation (B) UV irradiation and (C) Hydrogen peroxide. Wild-type *D. radiodurans* R1 (black square) was compared with the Mt-0865 strain (black circle). Error bars represent standard deviations from three replicate experiments.

The response of the Fur regulator to oxidative stress is very complicated in some microorganisms [5]. Because the Mt-0865 strain exhibits a growth defect and is sensitive to Mn stress, we further investigated the sensitivity of Mt-0865 to H_2_O_2_, UV and gamma irradiation. First, the survival of these strains was measured under oxidative stress. The results showed an increased sensitivity of Mt-0865 to H_2_O_2_, whereas the C-0865 strain exhibited a similar survival rate as the wild-type R1 strain (Figure 5 and Figure S4 in File S1). Furthermore, the survival rate was measured under UV and gamma irradiation. The D_10_ value, which represents the irradiating dose required to reduce the population by 90%, was used to assess the resistance of the wild-type R1 and Mt-0865 strains to gamma and UV irradiation. As shown in Figure 6A and B, the wild-type R1 strain showed higher resistance to gamma irradiation and UV radiation than the Mt-0865 strain. Similarly, the mutant strains show higher sensitivity to hydrogen peroxide, as shown in the Figure 6C

### Loss of *dr0865* results in an accumulation of intracellular Mn

It has been previously reported that a high intracellular Mn^2+^/Fe^2+^ ratio in *D. radiodurans* helps to protect proteins from oxidative damage, and contributes to its extreme resistance [15], [22]. Because the Mt-0865 is sensitive to Mn stress, H_2_O_2_ stress, as well as UV and gamma irradiation, inductively coupled plasma mass spectrometry (ICP-MS) analyses were performed to show whether the Mt-0865 strain had lost its ability to maintain homeostasis of manganese and other ions.

**Figure 7:**
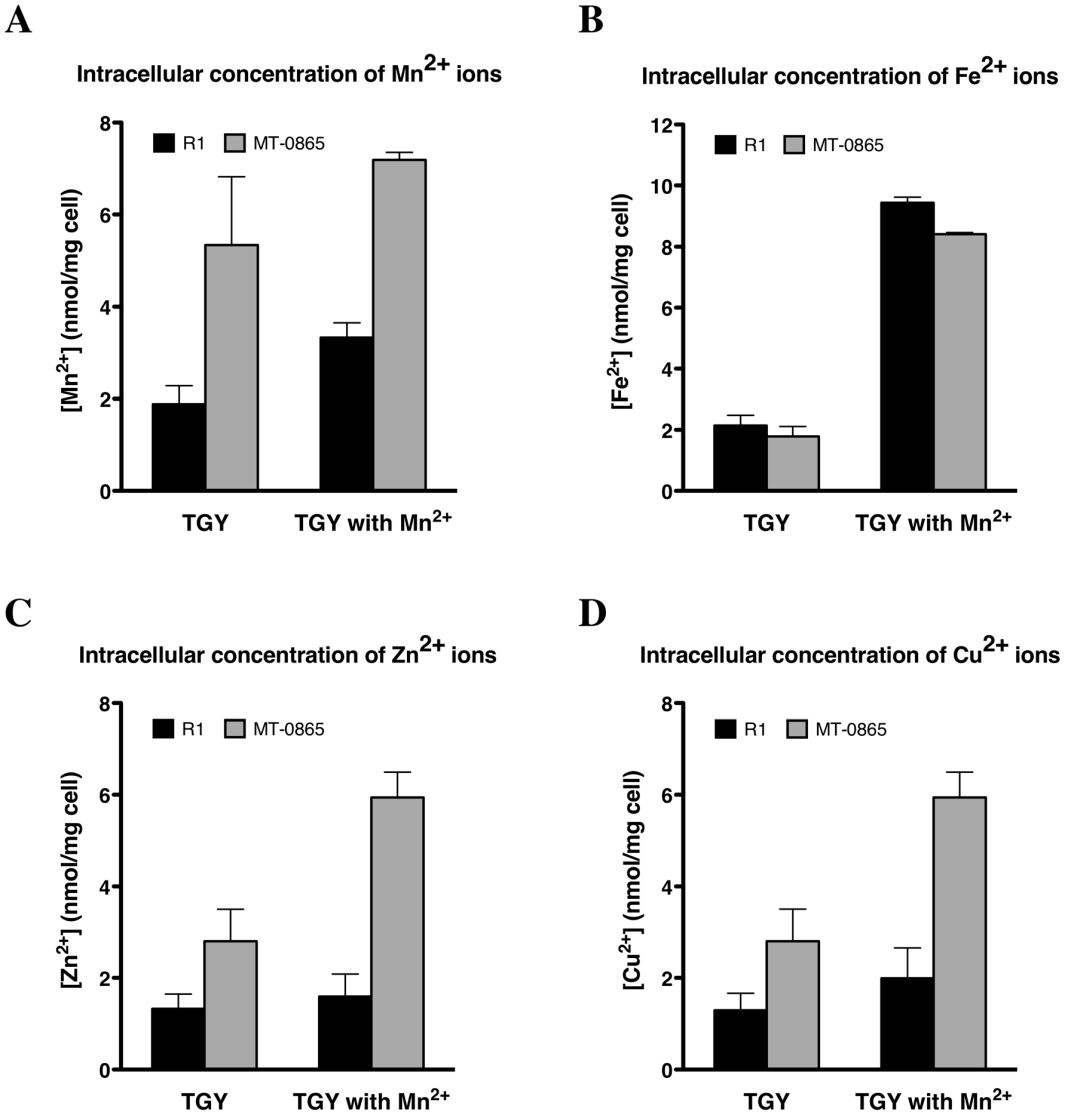
Analysis of the intracellular ion content of the wild-type R1 and Mt-0865 strains, cultured in a medium supplemented with or without 50 µM manganese; (A) manganese, (B) iron, (C) zinc, (D) copper. The data represent the mean ± standard deviation of three independent experiments.

As expected, even on TGY medium, the Mn (II) level in the Mt-0865 strain was almost three-fold higher than in the wild-type R1 strain (Figure 7A). Similar results were obtained when the two strains were grown on TGY medium supplemented with Mn^2+^. Furthermore, we also found that the Mn^2+^ level was increased when the wild-type R1 strain was grown on manganese-rich TGY medium, compared to normal medium (Figure 7A). In contrast, there was no significant difference in Fe^2+^ concentrations between the Mt-0865 and wild-type R1 strains. However, the Fe^2+^ concentrations in both wild type and mutant strains increased under Mn stress, indicating that *D. radiodurans* has a system to regulate Mn/Fe homeostasis (Figure 7B). Collectively, these results verified that the Mt-0865 strain is sensitive to Mn^2+^ stress and that the sensitivity of the mutant to damaging agents may be caused by excess manganese.

### Transcriptome changes in the *dr0865* mutant under manganese stress

Because DR0865 is homologous to transcriptional regulators and its disruption resulted in sensitivity to excess manganese, RNA sequencing (RNA-seq) was used to assess changes in transcripts of the wild-type R1 and Mt-0865 mutant strains when cultured in the presence of high (20 mM) levels of MnCl_2_. In total, 12.7 million (M) and 13.3 M pair-end reads were obtained for the wild-type R1 and Mt-0865 strains, respectively. After cleaning the reads, 9.3 M and 9.8 M reads mapped to the genome, and, after removing rRNA sequences, the unique sequence reads were 4.3 M and 4.4 M (Table 1).

**Table 1:** Summary of sequence reads statistics resulting from Illumina deep sequencing of mutant and wild type strain.

	Mutant Strain	Wild type strain
Raw data (read number)	13289865	12798354
Raw data (length, bp)	200	200
Clean data (read number)	10260088	9998560
Clean data (length, bp)	176	178
Mapping to genome (read number)	9831262	9299761
Mapping to rRNA	5289176 (53.8%)	4728594 (50.85%)
Unique mapping	4441705 (45.18%)	4387406 (47.18%)

A total of 3,098 of the 3,167 open reading frames (ORFs) of the wild-type R1 strain, and 3,060 of the 3,167 ORFs of Mt-0865 mutant strain were detected by RNA sequence data. In the Mt-0865 strain, 246 genes were up-regulated more than two-fold (Table S1 in File S1) and 317 genes were down-regulated more than two-fold in comparison to the wild-type R1 strain (Table S2 in File S1). The significantly expressed genes were classified in accordance with the Cluster of Orthologous Groups (COG) of proteins database (Table 2). The top three categories were transcription (22%), inorganic ion transport and metabolism (19.2%), and nucleotide transport and metabolism (18.5%).

**Table 2:** Classification of the genes with different levels of expression according to the Cluster of Orthologous Groups of proteins (COG) database.

COG_type^1^	Total genes	Induced genes	Repressed genes	Total rate^2^
**Information storage and processing:**				
J	165	10	6	9.6%
K	149	13	21	22.8%
L	138	5	12	12.3%
B	1	0	0	0
**Cellular processes and signaling:**				
D	26	0	4	15.4%
V	50	1	7	16%
T	119	5	9	11.2%
M	110	6	9	13.6%
N	21	0	2	9.5%
U	40	0	3	7.5%
O	108	6	12	16.7%
**Metabolism:**				
C	140	7	28	25%
G	114	6	10	14%
E	251	13	15	11.2%
F	92	10	7	18.5%
H	110	8	9	15.5%
I	99	5	3	8%
P	130	14	11	19.2%
Q	53	4	2	11.3%
**Poorly characterized:**				
S	251	19	23	16.7%
R	333	19	23	12.6%

1 . J: Translation, ribosomal structure and biogenesis; K: Transcription; L: Replication, recombination and repair; B: Chromatin structure and dynamics; D: Cell cycle control, cell division, chromosome partitioning; V: Defense mechanisms; T: Signal transduction mechanisms; M: Cell wall/membrane/envelope biogenesis; N: Cell motility; U: Intracellular trafficking, secretion, and vesicular transport; O: Posttranslational modification, protein turnover, chaperones; C: Energy production and conversion; G: Carbohydrate transport and metabolism; E: Amino acid transport and metabolism; F: Nucleotide transport and metabolism; H: Coenzyme transport and metabolism; Lipid transport and metabolism; P: Inorganic ion transport and metabolism; Q: Secondary metabolites biosynthesis, transport and catabolism; S: Function unknown; R: General function prediction only.

2 . the total number of significant genes/Number of total genes in this COG

In this study, our analysis focused on the genes involved in (i) Mn/Fe metabolism, (ii) ROS production, (iii) DNA damage response genes, and (vi) cell cleaning genes (Table 3).

**Table 3:** The significant genes were classified into three classes, Mn/Fe metabolism, ROS production genes, and Damage response genes.

ORF	Name	Description	M^1^
**Mn/Fe metabolism:**			
DR2283	*dr2283*	Mn ABC transporter permease	1.99
DR2284	*dr2284*	Mn ABC transporter permease	2.39
DR2523	*fimA*	Mn/Fe transport system substrate-binding protein	3.79
DR1236	*mntE*	manganese efflux protein	−1.69
DR1220	*feoA*	ferrous iron transport protein A	1.35
ROS production genes			
DR0342	*dr0342*	cytochrome complex iron-sulfur subunit	−1.45
DR0344	*ccmH*	cytochrome c-type biogenesis protein	−1.09
DR0346	*ccmF*	cytochrome c-type biogenesis protein	−1.09
DR0347	*ccmE*	cytochrome c-type biogenesis protein	−1.30
DR0348	*dr0348*	cytochrome c-type biogenesis heme exporter protein C	−1.46
DR2095	*dr2095*	c-type cytochrome	−1.39
DR2617	*ctaA*	cytochrome AA3-controlling protein	−1.11
DRC0001	*drc0001*	cytochrome P450-related protein	−∞^2^
DRC0041	*drc0041*	Cytochrome P450	−∞
DR1492	*dr1492*	NADH dehydrogenase I subunit N	−1.03
DR1493	*dr1493*	NADH dehydrogenase I subunit M	−1.68
DR1494	*dr1494*	NADH dehydrogenase I subunit L	−1.55
DR1497	*dr1497*	NADH dehydrogenase I subunit I	−2.54
DR1498	*dr1498*	NADH dehydrogenase I subunit H	−2.28
DR1499	*dr1499*	NADH dehydrogenase I subunit G	−1.57
DR1500	*dr1500*	NADH dehydrogenase I subunit F	−1.99
DR1501	*dr1501*	NADH dehydrogenase I subunit E	−1.76
DR1503	*dr1503*	NADH dehydrogenase I subunit D	−2.59
DR1504	*dr1504*	NADH dehydrogenase I subunit C	−2.32
DR1505	*dr1505*	NADH dehydrogenase subunit B	−1.79
DR1506	*dr1506*	NADH dehydrogenase I subunit A	−1.44
DRA0243	*Hmp*	Haemoglobin-like flavoprotein	−1.33
**Damage response genes:**			
DR1208	*Bcp*	Antioxidant type thioredoxin fold protein	∞
DR1209	*ahpC*	Thiol-alkyl hydroperoxide reductases	1.87
DRA0072	*grxA*	Glutaredoxin	3.38
DR2056	*hslJ*	Related to heat shock protein	1.18
DR0194	*htpX*	Predicted Zn-dependent proteases	1.69
DR0416	*mazE*	Regulatory protein, MazF antagonist	1.66
DR0417	*mazF*	ppGpp-regulated growth inhibitor	1.32
DR_B0088	*kdpD*	Osmosensitive K1 channel histidine kinasesensor domain	1.39
DR1667	*trkH*	Potassium uptake system component	−1.92
DR1678	*trkG*	Potassium uptake system component	−2.61
DRA0123	*arsC*	Arsenate oxidoreductase	−1.31
DR0455	*strA*	Streptomycin resistance protein	−1.62
DR2234	*dr2234*	involved in multidrug resistance	−2.41
DR1695	*gloA*	Lactoylgluthation lyase, fosphomicin resistance protein	1.49
DR0599	*BS_yokD*	amino glycoside N3-acetyltransferase	1.52
**Cell cleaning genes:**			
DR0092	*dr0092*	MutT/nudix family protein	2.69
DR0192	*dr0192*	MutT/nudix family protein	1.27
DR0261	*dr0261*	MutT/nudix family protein	3.33
DR0274	*dr0274*	MutT/nudix family protein	3.02
DR0784	*dr0784*	MutT/nudix family protein	1.18
DR0202	*clpX*	ATPase subunit of Clp protease	1.20
DR1974	*Lon*	ATP-dependent Lon serine protease	1.40
DR0958	*dr0958*	peptide ABC transporter permease	1.65
DR0959	*dr0989*	peptide ABC transporter permease	1.44
DR1358	*dr1358*	outer membrane protein	1.18
DRA0168	*dra0168*	ABC transporter permease	1.54
DRA0268	*dra0268*	adenine deaminase-like protein	1.21
DRA0323	*dra0323*	urea/short-chain amide ABC transporter ATP-binding protein	1.07

1 . M value means log_2_Ratio, Ratio = FPKM_(M-0865)_/FPKM_(R1)_

2 . ∞ means gene’s expression level is not detected in one sample, but detected in another sample.

### (i) Proteins involved in Mn^2+^ and Fe^2+^ metabolism

The expression of five genes involved in Mn/Fe metabolism was significantly changed under MnCl_2_ adaptation conditions (Table 2). *dr1236* (*mntE*), which encodes a putative manganese efflux family protein that controls the removal of excess Mn^2+^, was repressed (Table 2). However, all ATP-dependent Mn^2+^ transporter genes, including *dr2283*, *dr2284*, and *dr2523*, were induced, The Mn^2+^ transporter gene expression pattern suggested that Mn^2+^ concentration was increased in the mutant, which is in agreement with our ICP-MS data. Furthermore, a previous DNA binding assay also supported that ATP-dependent Mn^2+^ transporter genes and *mntE* are regulated by DR0865 [23].

### (ii) Proteins associated with the production of ROS

Manganese is among the essential enzyme cofactors because it protects cells from oxidative damage. However, it can be toxic at high concentrations and, therefore, its level should be strictly regulated [17], [23]. Previous research showed that cytochromes, flavoproteins, iron-sulfur proteins, NADPH and NADH-dependent enzymes are regarded as the major generators of ROS [1], [2]. Under Mn^2+^ stress, nine cytochrome-related genes and 12 NADH-dependent enzymes were repressed in the Mt-8065 mutant compared to the wild-type R1 strain. This indicates that the mutant strain compensates for its Mn sensitivity by dampening the production of ROS.

### (iii) Proteins associated with the DNA damage response

There were 15 induced genes associated with stress responses, but these did not include *katE* or *recA*. However, three antioxidant proteins, *bcp (dr1208)*, *ahpC (dr1209)*, and *grxA* (*dr1209*, *dra0072*), were up-regulated in the Mt-0865 strain (Table 3), which indicates that the mutant strain may be under oxidative stress. In addition to the well-characterized components of stress response systems, *D. radiodurans* encodes several proteins whose specific roles are unknown but are likely to be important for the multiple stress resistance phenotypes of the bacterium. An example of a poorly studied, but potentially important, system is the “addiction module” response, which is encoded by two genes, *mazE* (*dr0417*) and *mazF* (*dr0416*). MazF is a stable protein that is toxic to bacteria, whereas MazE protects cells from the toxic effect of MazF, and is degraded by the ClpX serine protease (*dr0202*) (Table 3). When the Mt-0865 mutant was under Mn stress, all of these genes were induced, which suggests that the mutant strain activates the antidote-toxin system to reduce cell growth to avoid the production of ROS. This result is also consistent with the expression patterns of ROS generating genes.

### (iv) Cell cleaning proteins

When the Mt-0865 mutant strain was under Mn stress, we found that the cellular cleansing system, including the export of damaged DNA components and sanitization of intracellular mutagenic precursors, was also induced. First, it was observed that six ABC transporter permease genes, which may control oligonucleotide export, were activated (Table 3) [24]. The export of damaged nucleotides outside the cell might protect the organism from elevated levels of mutagenesis by preventing the reincorporation of damaged bases during DNA synthesis [25]. Second, 15 of 20 *mutT/nudix* family genes were induced, five (*dr0092*, *dr0192*, *dr0261*, *dr0274*, dr0784) of which were up-regulated significantly (Table 3). The MutT protein has an 8-oxo-dGTPase activity, which can limit mutation of DNA by hydrolyzing the oxidized products of nucleotide metabolism. The remaining intracellular mutagenic precursors could be sanitized via this superfamily [26]. Finally, it was also found that Lon protease (DR1974) and ClpX protease (DR0202) were induced approximately twofold (Table 3). These ATP-dependent proteases help with cellular sanitization by degrading damaged proteins [27].

### qRT-PCR analysis

To confirm the transcriptome assay results, gene expression in the Mt-0865 mutant and in the wild-type R1 strains was analyzed using quantitative real time PCR (qRT-PCR) analysis. Eight genes (*dr2523*, *dr2283*, *dr1709*, *dr1236*, *dr1998*, *dr1506*, *dr0348*, and *dr0828*) were quantified under normal growth conditions and after treatment with Mn^2+^. Four of these genes are Mn^2+^ transport genes (*dr2523*, *dr2283*, *dr1709* and *dr1236*). The DR2523 and DR2283 proteins are ATP-dependent transporters, the DR1709 protein belongs to the Nramp family of transporters, and DR1236 is a Mn^2+^ efflux gene. The *dr1998* gene encodes a major catalase (KatE), which plays an important role in the protection of *D. radiodurans* from oxidative stress and ionizing radiation [1]. The DR1506 protein is a NADH dehydrogenase and DR0348 is the cytochrome c-type biogenesis heme exporter protein C. It was previously shown that *dr1506* and *dr0348* are associated with the production of ROS [1]. The *dr0828* gene encodes an isocitrate lyase, which is an enzyme in the glyoxylate cycle that catalyzes the cleavage of isocitrate to succinate and glyoxylate. Previous research has shown that when irradiated, *D. radiodurans* represses the tricarboxylic acid (TCA) cycle and activates the glyoxylate bypass [28].

**Figure 8:**
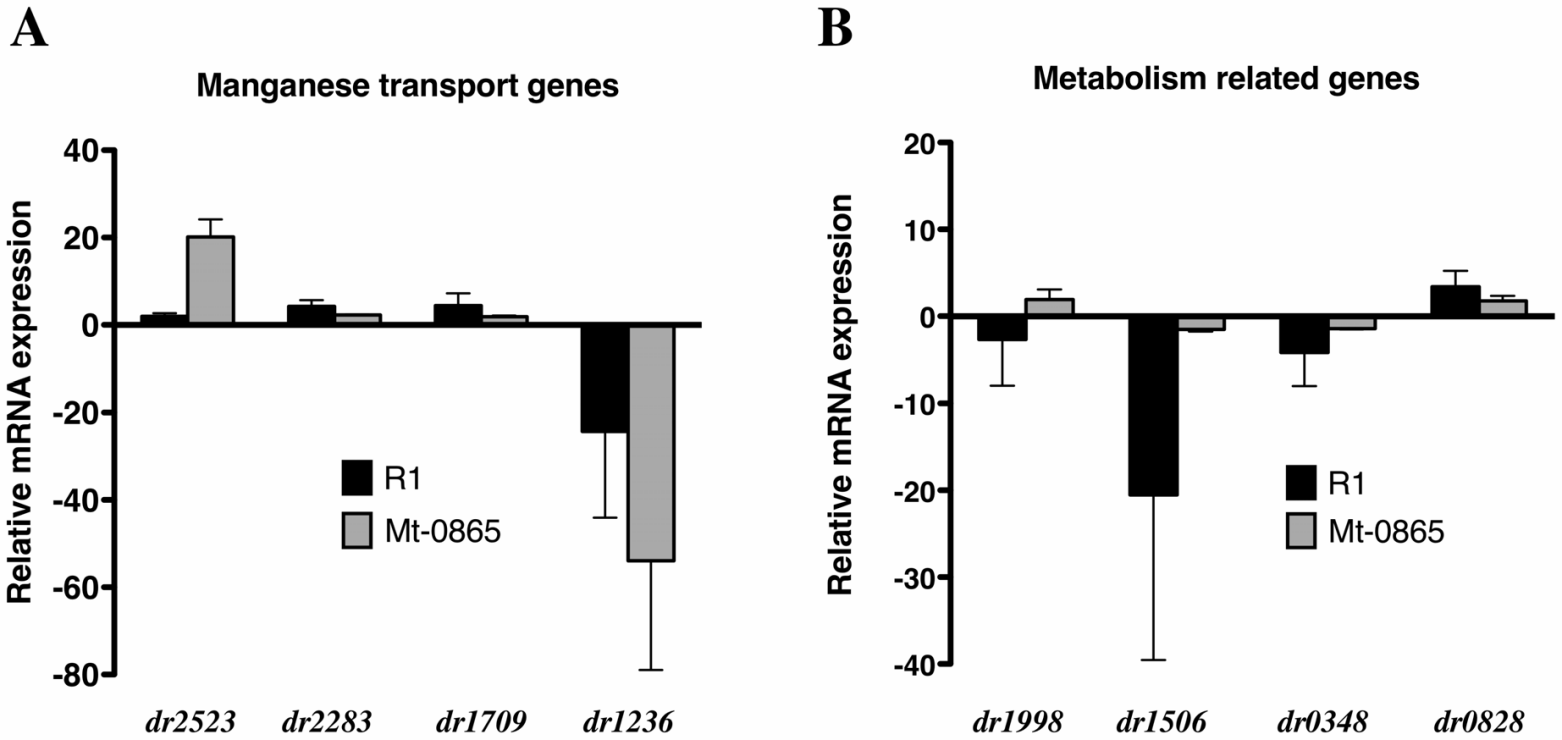
The expression of potential DR0865-dependent genes in wild-type *D. radiodurans* compared to the mutant strain, under normal conditions (black bar), and wild-type R1 under manganese stress compared with the mutant strain under manganese stress (grey bar). Error bars represent standard deviations from three replicate experiments. (A) Four manganese transport genes, (B) metabolism related genes.

It was observed that under normal growth conditions, the expression of *dr2523*, *dr2283*, and *dr1709* increased 1.96-fold, 4.24-fold and 4.41-fold, respectively, in the Mt-0865 mutant strain compared to the wild-type R1 strain (Table S3 in File S1), while the *dr1236* gene was significantly repressed 24.32-fold in the mutant strain. In addition, the transcript levels of *dr1506* and *dr0348* decreased, whereas the level of the *dr0828* transcript increased, in the mutant strain (Figure 8A and Table S3 in File S1). Gene expression levels were also measured under Mn^2+^ stress. The expression of the Mn^2+^ transporter gene *dr2539* increased 20.14-fold, while expression of *dr1236* decreased 53.94-fold in the Mt-0865 mutant (Figure 8A). This suggests that under Mn^2+^ stress, the wild-type strain attempts to stop Mn^2+^ uptake and opens the Mn^2+^ efflux system, whereas this does not occur in the mutant strain. These results provide further evidence that Mn^2+^ transporter genes are not properly expressed in the mutant. In addition, the *dr1506* and *dr0348* genes were repressed and the *dr0828* gene was induced. This suggests that, under Mn (II) stress, the wild-type strain lowers its metabolic rate to reduce ROS production and activates the glyoxylate bypass to provide energy, whereas these adaptations are defective in the mutant strain. Overall, the pattern of gene expression indicates that the mutant strain is likely subject to more damage than the wild-type strain under Mn^2+^ stress.

## Discussion

Manganese is a trace element that is essential for many cellular functions in all organisms. For example, Mn^2+^ is required as a co-factor for super-oxide dismutase, which is critical for preventing cellular oxidative stress [29]. However, high manganese levels inhibit calcium influx and promote the exchange of accumulated Ca^2+^, and inhibit RNA and protein synthesis [3]. Thus, maintaining metal ion homeostasis is necessary for all organisms. *D. radiodurans* is well known for its extreme resistance to radiation and oxidants and its high intracellular Mn/Fe ratio is an important factor that contributes to this resistance. In this study, we identified a unique Mur homolog that is encoded by *dr0865*, and data showed that it is Mn^2+^-specific regulator.

Sequence analyses showed that DR0865 contains three metal-binding domains that are present in the *H. pylori* Fur homolog. Previous data showed that the C_92_XXC_95_ motif is necessary for the construction of the ZnS_2_ (N/O)_2_ domain, while the C_133_XXXXC_138_ motif is not important [20], [30]. Further research is needed to discern the function of the C_82_XXC_85_ and C_112_XXC_115_ motifs in *D. radiodurans.* Because the phenotype of the Mt-0865 mutant strain showed that DR0865 is a novel Mur protein, we compared the DR0865 amino acid sequence to the *R. leguminosarum* Mur protein [12]. The results showed that the *R. leguminosarum* Mur protein does not have domain I, while it contains domains II and III (data not shown). This indicates that domains II and III are important for Mn^2+^ ion regulation.

It has been suggested that the accumulation of Mn^2+^ or a higher Mn/Fe ratio benefit the radio-resistance of *D. radiodurans*. However, the excess of Mn^2+^ is toxic to the cell. Although the precise mechanism of Mn^2+^ toxicity is poorly understood, three mechanisms have been suggested previously. In the first mechanism, Mn^2+^ cell toxicity may be associated with its interaction with other essential trace elements, such as Fe^2+^, Zn^2+^ and Cu^2+^ [31]. Human studies have shown that chronic exposure to Mn^2+^ appears to be associated with similar increases in cellular Fe^2+^ uptake, which consequently produces cellular oxidative stress and also increases the concentration of Cu^2+^ and Zn^2+^ [31]. When the wild-type R1 strain was under Mn^2+^ stress, the intracellular concentration of Mn^2+^ and Fe^2+^ increased significantly, whereas the concentrations of Zn^2+^ and Cu^2+^ increased slightly (Figure 7C and D). Under normal growth conditions, the Mt-0865 strain had higher Mn^2+^, Cu^2+^ and Zn^2+^ contents than the wild-type R1 strain. These data further confirmed that mutation of the *dr0865* gene causes a defect in the control of Mn^2+^ metabolism, which also results in changes in the concentrations of Cu^2+^ and Zn^2+^. Interestingly, Fe^2+^ concentration was not significantly different between the wild-type and mutant strains under Mn^2+^ stress (Figure 7B), which may be due to the distinct *D. radiodurans* Fe^2+^ regulation system, which utilizes OxyR and DtxR regulators.

The second mechanism is that high intracellular levels of Mn^2+^ inhibit RNA and protein synthesis, and manganese may exert a toxic effect through such inhibition [3]. When *Bacillus stearothermophilus* was grown in media containing excess Mn^2+^, its doubling time increased more than two-fold. A similar effect on growth was also observed in the Mt-0865 mutant under normal growth conditions. The third mechanism suggests that Mn^2+^ can participate in reactions that potentially increase ROS, which subsequently causes oxidative damage [32]. These three mechanisms may explain why the mutant was sensitive to different DNA damaging agents.

The RNA-seq data identified 562 genes (approximately 17% of the genome) that showed at least a twofold change in expression between the Mt-0865 mutant and the wild-type R1 strain, which indicates that these genes were regulated by *dr0865* either through direct or indirect mechanisms. Using the Kyoto Encyclopedia of Genes and Genomes (KEGG) database, we found that genes involved in metabolic pathways, the biosynthesis of secondary metabolites, oxidative phosphorylation and nitrogen metabolism were significantly repressed in the mutant strain. This indicates that the Mt-0865 mutant is likely to suffer more cellular damage under Mn^2+^ stress than the wild-type strain. This phenomenon may be caused by higher Mn^2+^ levels in the mutant, which would increase ROS levels and lead to DNA damage [33]. Five *mutT/nudix* family genes were also activated but no major DNA repair genes (such as *recA* or *pprA*) were induced, which further confirms our hypothesis.

Interestingly, we found that the heme biosynthesis pathway (HemA, HemE and HemN) was slightly repressed in the Mt-0865 mutant (Table S2 in File S1). Hemes are biosynthesized from protoporphyrin and free ferrous iron [34] and are cofactors for cytochromes, catalases and peroxidases. This may explain why nine cytochrome genes were down-regulated under Mn^2+^ stress in the mutant. In addition, two vitamin B12 biosynthesis proteins, *drb0010* (cobalamin biosynthesis protein) and *drb0012* (cobyric acid synthase), were also repressed. Vitamin B12 is a water-soluble vitamin that is normally involved in DNA synthesis and regulation, as well as in fatty acid biosynthesis and energy production. The *dr0910* and *dr1076* genes, which encode cell wall protein and cell wall synthesis proteins, respectively, were also down-regulated. The reduction of vitamin B12 and cell wall proteins may be caused by high Mn (II) levels, which consequently results in the inhibition of cell growth.

Five ribonucleases (*dr0020*, *dr0859*, *dr1949*, *dr2374* and *drb0107*) were induced at least two-fold in the Mt-0865 mutant (Table S1 in File S1). Ribonucleases in prokaryotic toxin-antitoxin systems are proposed to function as stress-response elements. The degradation of RNA within a cell leads to fragments of RNA that are no longer needed and can be cleaned up as part of the cellular protection system. Five cation transporter genes (*dr0748a*, *dr0816*, *dr0883*, *dra0168*, *dra0361*) were also activated, which explains why the mutant had higher ion concentrations.

Overall, our work presents a biochemical mechanism for Mn (II) sensing by the Mur homolog gene in *D. radiodurans*. Using qRT-PCR and global transcriptome analysis, we provided evidence that DR0865 functions as a positive (*dr1236*) and a negative regulator (*dr2283*, *dr2284*, *dr2523*) of different classes of Mn^2+^ transporter genes. More research is needed to establish the detailed mechanism of Mur regulation of these important genes. The potential communication between OxyR and other regulators, such as DtxR (dr2539), should be explored to determine whether it is required for the intricate coordination of oxygen radical detoxification.

## Experimental Procedures

### Strains, media and primers

All the primers used in this study are listed in Table 4. The *E. coli* strains were grown in Luria-Bertani (LB) broth medium (1% tryptone, 0.5% yeast extract and 1% sodium chloride) with aeration or on LB agar plates (1.2% Bacto-agar) at 37°C supplemented with the appropriate antibiotics. All *D. radiodurans* R1(ATCC 13939) strains used in this work were grown at 30°C in TGY medium (0.5% tryptone, 0.1% glucose and 0.3% yeast extract) with aeration or on TGY plates supplemented with 1.5% Bacto-agar.

**Table 4:** Bacterial strains and plasmids used in this study.

Strain or plasmid	Relevant marker	Source
**Strains**		
*E. coli* DH5α	Propagation for plasmid	Invitrogen
*E. coli* BL21(pLysS)	DR0865 expression strain	Invitrogen
*D. radiodurans* R1	ATCC13939	This lab
Mt-*0865*	As R1, but *dr0865*::*kan*	This study
C-0865	Mt-*0865* complemented with *pRKR*	This study
**Plasmids**		
pMD18-T	TA cloning vector	Takara
pMR	pET-28a derivative recombinant expressing and dr0865	This study
pRADK	*E. coli-D*. radiodurans shuttle vector carrying *D.radiodurans gro*EL promoter	[36]
pRKR	pRADK derivative expressing *D.radiodurans* dr0865	This study

### Sequence alignment

The protein sequence of previously characterized Fur proteins found in *A. ferrooxidans*, *P. aeroginosae*, *E. coli, B. subtilis, M. marinum, D. radiodurans*, *H. pylori* were obtained from the NCBI database. The protein sequence alignment of selected Fur proteins was generated using ClustalW.

### Disruption of the DR0865 gene in *D. radiodurans*

The mutant strain was constructed as described previously [18]. Primer ME1 and ME2 were used to amplify a *Bam*HI fragment upstream of targeted genes, and primers ME3 and ME4 were used to obtain a *Hin*dIII fragment downstream of targeted genes respectively (Table 5). The kanamycin resistance cassette containing the *gro*EL promoter was obtained from a shuttle plasmid, pRADK. After this three DNA fragments were digested and ligated. The ligation products were used as template for PCR to amplify the resulting PCR fragment (ME1 and ME5 used as primers), which was then transformed into exponential-phase cells by CaCl_2_ treatment [35]. The mutant strains were selected on TGY agar plates supplemented with 30 µg/ml kanamycin. Null mutants were confirmed by PCR product sizes, enzyme-digested electrophoresis, and DNA sequencing and the resulting mutant was designated Mt-0865.

**Table 5:** Primers used in this study.

Primer	Sequence (5′ → 3′)
**Mutation primers**	
0865upF(ME1)	CGAAGAAGTCGCCAACAACC
0865upR(ME2)	GGATCCGGAGGCAGGGTAGCAAAGCG
0865downF(ME3)	AAGCTTGGCGGGAAGTTTTTACTGCGTG
0865downR(ME4)	ACACTAACCGTTTTTCGCCATTGCC
**Complement primers**	
0865F (ME5)	CATATGACCGCCCGCCGCAGCAC
0865R (ME6)	GGATCCTTAGTGGGCCCCGGTCTTC
**Real time PCR primers**	
RT-dr1506F	GCGGGAAAGGCTGGAGTCAGGAGG
RT-dr1506R	CTTGGTGCGGGTCGCCTTTTTGGG
RT-dr0348F	CCTCGGGTACTTCATCATCCGTGGC
RT-dr0348R	TTGACGGTGGCGGTCTGGTGAATG
RT-dr1236F	CATCAATCTGGTGTGGGCGAAC
RT-dr1236R	CAAGCAGCGGGTCAAGGATGTG
RT-dr0828F	GACACCATGACCCCCACCCCCAAAA
RT-dr0828R	GGTGTACTCGATGGGCAGGCTG
RT-dr2523F	CGACGCCCATACCTTTCAGC
RT-dr2523R	GTCAGCTCCTTCACCGGCAC
RT-dr2283F	GGAGCCTGCGGACCATGA
RT-dr2283R	GCGAGCGCCAGCAGAAAA
RT-dr1998F	GGGCGTGGACAAGCGTATTC
RT-dr1998R	GTAGACGGGGGCTTCCTGCT
RT-dr1709F	GCGATGGTGATTCAGAACCT
RT-dr1709R	GTTCGGCCTGAATCCAGTAA

**Note**: straight line represents restriction site.

### Complementation of DR0865 mutant

Complementation strain was constructed as described [18], [36]. Briefly, genome DNA was isolated from wild-type R1 strain. A 2500-bp region containing the *dr0865* gene was amplified by ME5 and ME6 (Table 5), and ligated to pMD-18 T-Easy vector (Takara, JP), designed as pMD-dr0865. After digested by *Nde*I and *Bam*HI, the target gene *dr0865* was ligated to *Nde*I and *Bam*HI-pre-digested pRADK, which named as pRKR. The complementation plasmid were confirmed by PCR and DNA sequence analysis, and transformed into Mt-0865, resulting in functional complementation strains. Selection for *D. radiodurans* complement strain was achieved on TGY plates, supplemented with kanamycin (30 µg/ml) and chloramphenicol (3 µg/ml).

### Growth curve assay

To examine bacterial growth in vitro as described previously was little modified [37], the single clone of the wild-type R1, Mt-0865 and C-0865 strains were transferred into 5 ml liquid TGY medium. When the OD_600_ of the cultures reached 1.0, 1 mL of each culture was added to 100 mL fresh TGY medium. Three repeats were performed for each strain. The nine cultures were incubated with shaking at 30°C and samples were taken every two hours to measure the OD_600_ value. The cultures were incubated with 250 rpm at 30°C and samples were taken to measure the OD_600_ value at different time. All experiments were repeated in triplicate.

### Cation sensitivity assays

Cation sensitivity assays were carried out as described previously [17]. Solutions (1 M) of manganese chloride, manganese sulfate, zinc chloride, copper chloride, copper sulfate, cobalt (II) chloride, nickel chloride, cadmium chloride, ferrous sulfate, ferrous chloride, ferric chloride, magnesium chloride, calcium chloride (sigma) were prepared in milli-Q water and filter-sterilized by passing through 0.22-µm filters. Newly fresh clone was taken from the wild-type R1, Mt-0865 and C-0865 TGY plates, into 5 ml TGY fresh media, when the cells grown up to stationary phase. Then, the cells were plated on TGY plates and overlaid with 5-mm sterile discs containing 1 M various cation solutions. The plates were incubated for three days, and the inhibition zone of each disc was measured. All the data provided here represent the mean and standard deviation of at least three independent experiments (mean ± SD of three experiments).

Similarly, to ascertain the effect of Mn^2+^ on growth of Mt-0865 and wild-type R1, 1×10^5^ CFU ml^−1^, were grown in TGY supplemented with increasing concentration of MnCl_2_. The OD_600_ value was measured after 12 h and 24 h post incubation. All the data provided here represent the mean and standard deviation of at least three independent experiments (mean ± SD of three experiments).

### H_2_O_2_ sensitivity assays (Oxidative stress assays)

The discs diffusion assay to test H_2_O_2_ sensitivity, was performed as described previously with a little modification [38], [39]. The strain was cultured up to log phase and 130 µl aliquots were spread on TGY plates. A sterile 5 mm-diameter filter discs, containing 4 µl and 6 µl of 1 M H_2_O_2_ was placed on the surface of the TGY plate. After incubation at 30°C for three days, the size of the area cleared of bacteria (zone of inhibition) was measured. For the curve H_2_O_2_ treatment, the cultures were treated with different concentrations of H_2_O_2_ for 30 min and then plated on TGY plates, as prescribed previously [40]. All the data provided here represent the mean and standard deviation of at least three independent experiments (mean ± SD of three experiments).

### Gamma irradiation and UV sensitivity assays

Survival curves of the wild-type R1 and Mt-0865 cells were cultured in TGY broth to OD_600_ ∼1.0. For the Gamma radiation treatment, the 100 ml cultured was irradiated with different doses of ^60^Co gamma at room temperature, which correspond to doses from 0 to 16 kGy, as previously published [41], [42]. After the irradiation treatment, the culture centrifuged and then re-suspended in phosphate buffer (1XPBS Buffer, pH 7.5). The cells were plated on TGY plates and incubated at 30°C for at least three days. The colonies were counted. All the data provided here represent the mean and standard deviation of at least three independent experiments (mean ± SD of three experiments).

For the UV treatment, the cells were cultured in TGY broth to OD_600_∼1.0, as described previously [17], [43]. The cells were re-suspended in 1XPBS buffer (pH 7.5), then plated on TGY plates and exposed to different doses of UV radiation at 254 nm. All the data provided here represent the mean and standard deviation of at least three independent experiments (mean ± SD of three experiments).

### Assay of intracellular Mn, Fe, Zn and Cu ion concentration

The protocol for determining intracellular concentration of metals ions was identical to previously reports [17]. *D. radiodurans* R1 and Mt-0865 were cultured in 5 ml TGY broth and re-inoculated in 500 ml TGY broth which had been pretreated with Chelex to remove any cat-ion, and then supplemented with 50 µM manganese chloride. The cells were grown up to OD_600_ ∼0.6–0.8 and harvested. After centrifugation at 10000 g, 4°C for 10 min, the pellets were washed three times with 1xPBS (pH 7.5), containing 1 mM EDTA and rinsed three times with 1xPBS, without EDTA. Cells (1/10 of the total volume) were withdrawn to measure the dry weight. For ion analysis, 1 ml of Ultrex II nitric acid (Fluka AG., Buchs, Switzerland) was added to the rest cells and incubated at 100°C for 1 h. After centrifugation at 20,000 g for 20 minutes, the supernatant was filtered against 0.45 µM membrane. The concentration of samples was analyzed for ion content by inductive coupled plasma mass spectrometry (ICP-MS, Model Agilent 7500a, Hewlet-Packard, Yokogawa Analytical System, Tokyo, Japan). A control prepared in the same manner but without 50 µM manganese chloride. All the data provided here represent the mean and standard deviation of at least two independent experiments (mean ± SD of twice experiments).

### Total RNA isolation

To see the effect of Mn^2+^ on the genome level, the total RNA was extracted from the three biological replicates of wild-type R1 and Mt-0865 under Mn^2+^ stress. Briefly, the wild-type R1 and Mt-0865 strains were cultured in a 5 ml TGY broth and re-inoculated in a 500 ml TGY broth. When the cells grow to OD_600_∼0.4–0.45, 20 mM MnCl_2_ was added to the broth and further cultured at 30°C for half an hour. The pellets were washed three times with 1XPBS buffer (pH 7.5), and total RNA was extracted from cell cultures using TRIzol reagent (Invitrogen US, ice) as the kit protocol.

### Bacterial RNA sequence library construction

Total RNA from three wild-type R1 and mutant strain (Mt-0865) were pooled, respectively, and rRNA (include 16S and 23S) was removed from 4 µg total RNA by MicrobexpressTM (Ambion AM1905), and the left RNA was chemically fragmented. The sequence library construction is according to ScriptSeq mRNA-Seq Library Preparation Kit (Illumina-compatible). Briefly, the fragmented RNA is reverse-transcribed into cDNA using the SuperScript double-stranded cDNA synthesis kit (Invitrogen) with the addition of SuperScript III reverse transcriptase (Invitrogen), and random primers containing a tagging sequence at their 3’ends. This was followed by RNase A (Roche, Germany) treatment, phenol-chloroform extraction, and ethanol precipitation. The resulting cDNAs were ligated to 5’ DNA/DNA adaptor, and the di-tagged cDNAs was purified by PAGE gel, the insert fragment size is 150 bp∼250 bp. The purification products were PCR amplified in 18 cycles using a high-fidelity DNA polymerase. PCR products were purified using the PAGE gel. Both direct cDNAs were sequenced simultaneously using a single flow cell of the Illumina Hiseq2000. All the sequence assays were performed in Zhejiang TianKe Company.

### Transcriptome analysis

The images generated by the sequencers were converted into nucleotide sequences by a base-calling pipeline. The raw reads were saved in the fastq format. Three criteria were used to filter out the raw reads according to previously published [44], (i) Remove reads with sequence adaptors; (ii) remove reads with more than 20% ‘N’ bases; (ii) remove low-quality reads, which have more than 40% QA ≤20 bases. All subsequent analyses were based on clean reads. Only reads with high quality value were selected and used in the mapping using Tophat [45]. No more than 2-mismatches were allowed in the alignment for each read, and only the unique mapping reads used in the latter analysis. Cufflink and Cuff-diff were used to calculate Fragments Per Kilo base of transcript per Million mapped reads (FPKM), and find significantly expressed genes, respectively. The annotation of the *D. radiodurans* genome obtained from NCBI.

### Reverse transcription-PCR (RT-PCR) analysis of expression of genes

QRT-PCR assay utilized RNA samples obtained from different condition and first-strain cDNA synthesis was carried out in 20 µl of reaction containing 1 µg of RNA sample combined with 3 µg of random hexamers using SuperScript III Reverse Transcriptase kit (Invitrogen). Each measurement was obtained for three replicate. Then Quant SYBER Premix FX TaqTM (TaKaRa Biotechnology (Dalian) Co. Ltd, China) was used to amplification following the manufactures instruction. As an internal control, *dr0089* was used as a house-keeping, encoding the glycosyl transferase [18]. All primers used in QRT-PCR are shown in Table 5. All assays were performed using the STRAGENE Mx300PTM Real-time detection. Data analysis was carried out with iCycler software (Bio-rade Laboratories). The ratio of the copy number for the treatment to the control copy number was calculated. Differences in relative transcript abundance level were calculated using 2^−ΔΔT^ [18].

### Statistical analysis

All data are presented as mean ± standard error of the mean (SEM). Statistical analysis was performed on the raw data using paired student’s *t*-test; *p* values <0.05 were considered significant.

## Supporting Information

File S1 **Combined Supporting Information file.**
(DOC)
