# Supplementary material for: A Mur Regulator Protein in the Extremophilic Bacterium Deinococcus radiodurans
Source: PLoS One. 2014 Sep 22;9(9):e106341. doi: 10.1371/journal.pone.0106341 (PMC4171365; doi:10.1371/journal.pone.0106341)
Supplement: File S1 — Combined Supporting Information file. (DOC) [file pone.0106341.s001.doc]

Table S1: The induced genes showed in Mt-0865. All genes are sorted by gene id.

| **Synonym** | **R1** | **Mt0865** | **fold_change** | **COG** | **GO** | **KO** | **Product** |
| --- | --- | --- | --- | --- | --- | --- | --- |
| DR_0016 | 24.0633 | 66.9715 | 1.47671 | COG0846K | GO:0070403 | K12410:ko00520 Amino sugar and nucleotide sugar metabolism | hypothetical protein |
| DR_0017 | 13.6659 | 30.8132 | 1.17297 | COG3877S |  |  | hypothetical protein |
| DR_0020 | 17.1594 | 45.1415 | 1.39546 | COG0557K | GO:0004540 | K01147: | ribonuclease II family protein |
| DR_0092 | 2.7006 | 17.5256 | 2.69812 | - | GO:0016787 |  | MutT/nudix family protein |
| DR_0125 | 219.157 | 544.193 | 1.31216 | COG1247M | GO:0016740 |  | acetyltransferase |
| DR_0133 | 2.98102 | 8.48862 | 1.50972 | COG0657I | GO:0016787|GO:0008236 | | lipase/esterase |
| DR_0175 | 120.818 | 258.244 | 1.0959 | COG0103J | GO:0030529 | K02996:ko03010 Ribosome | 30S ribosomal protein S9 |
| DR_0192 | 19.6554 | 47.3789 | 1.26932 | COG1051F | GO:0016787 | K03574: | MutT/nudix family protein |
| DR_0193 | 13.7951 | 59.2236 | 2.10201 | COG2383S |  |  | hypothetical protein |
| DR_0194 | 671.39 | 2170.24 | 1.69263 | COG2738R |  | K06973: | hypothetical protein |
| DR_0202 | 20.7092 | 47.5716 | 1.19983 | COG0563F | GO:0016310 |  | hypothetical protein |
| DR_0206 | 9.60431 | 22.1936 | 1.20839 | - |  |  | hypothetical protein |
| DR_0230 | 17.2382 | 60.9146 | 1.82118 | COG0245I | GO:0046872 | K01770:ko00900 Terpenoid backbone biosynthesis | hypothetical protein |
| DR_0241 | 3.11954 | 17.5127 | 2.48899 | - |  |  | hypothetical protein |
| DR_0259 | 8.06968 | 19.6301 | 1.28249 | COG1846K | GO:0006355 |  | hypothetical protein |
| DR_0261 | 1.9867 | 20.0403 | 3.33445 | COG1051F | GO:0016787 | K03574: | MutT/nudix family protein |
| DR_0273 | 14.4611 | 40.5723 | 1.48832 | COG2258S | GO:0030170 |  | hypothetical protein |
| DR_0274 | 2.21885 | 17.9515 | 3.01623 | - | GO:0016787 |  | MutT/nudix family protein |
| DR_0296 | 40.0641 | 93.7618 | 1.22669 | - |  |  | hypothetical protein |
| DR_0301 | 17.8062 | 39.7777 | 1.15958 | COG2331S |  |  | hypothetical protein |
| DR_0302 | 56.6169 | 128.645 | 1.18409 | COG0449M | GO:0030246 | K00820:ko00250 Alanine, aspartate and glutamate metabolism | glucosamine--fructose-6-phosphate aminotransferase |
| DR_0319 | 561.715 | 1153.89 | 1.0386 | COG0255J | GO:0030529 | K02904:ko03010 Ribosome | 50S ribosomal protein L29 |
| DR_0338 | 145.972 | 352.795 | 1.27314 | - |  |  | hypothetical protein |
| DR_0402 | 38.7201 | 79.8343 | 1.04393 | COG1310R | GO:0008233 |  | hypothetical protein |
| DR_0416 | 73.013 | 231.343 | 1.66381 | COG2336T |  | K07172: | ppGpp-regulated growth inhibitor suppressor ChpR/MazE |
| DR_0417 | 12.0538 | 30.0206 | 1.31646 | COG2337T | GO:0003677 | K07171: | ppGpp-regulated growth inhibitor ChpA/MazF |
| DR_0418 | 112.347 | 302.168 | 1.42739 | - |  |  | hypothetical protein |
| DR_0420 | 271.133 | 624.046 | 1.20265 | COG1643L | GO:0016787 | K03579: | ATP-dependent helicase |
| DR_0431 | 46.069 | 107.948 | 1.22847 | COG2227H | GO:0032259 |  | hypothetical protein |
| DR_0443 | 23.8525 | 59.413 | 1.31664 | COG0213F | GO:0016763 | K00756:ko00240 Pyrimidine metabolism |K00758:ko00240 Pyrimidine metabolism | pyrimidine-nucleoside phosphorylase |
| DR_0449 | 24.9334 | 58.8452 | 1.23885 | - | GO:0004523 |  | hypothetical protein |
| DR_0451 | 63.9427 | 142.59 | 1.15702 | COG0174E | GO:0006807|GO:0016874 | K01915:ko00250 Alanine, aspartate and glutamate metabolism | glutamine synthase |
| DR_0452 | 582.261 | 1518.04 | 1.38247 | - |  |  | hypothetical protein |
| DR_0478 | 133.72 | 296.981 | 1.15116 | COG0006E | GO:0016787 |  | peptidase-like protein |
| DR_0492 | 20.6982 | 102.487 | 2.30787 | COG1359S | GO:0004497 |  | hypothetical protein |
| DR_0523 | 1.30496 | 3.94776 | 1.59702 | - |  |  | hypothetical protein |
| DR_0525 | 2.11303 | 5.97641 | 1.49996 | - |  |  | hypothetical protein |
| DR_0527 | 3.12559 | 19.686 | 2.65497 | - |  |  | hypothetical protein |
| DR_0529 | 18.452 | 52.571 | 1.51049 | - |  |  | hypothetical protein |
| DR_0567 | 5.94849 | 61.0659 | 3.35977 | COG1171E | GO:0009097 | K01754:ko00260 Glycine, serine and threonine metabolism | threonine dehydratase |
| DR_0590 | 57.9549 | 139.277 | 1.26496 | - |  |  | hypothetical protein |
| DR_0593 | 54.1277 | 123.707 | 1.19248 | COG0596R | GO:0016787 |  | beta-ketoadipate enol-lactone hydrolase |
| DR_0599 | 13.2434 | 38.0479 | 1.52254 | COG2746V | GO:0046677 |  | aminoglycoside N3-acetyltransferase |
| DR_0610 | 0 | 25.2119 | 1.79769e+308 | - | GO:0016310|GO:0016740 | | hypothetical protein |
| DR_0679 | 10.8562 | 33.1966 | 1.61251 | COG1669R | GO:0016779 | K07075: | hypothetical protein |
| DR_0685 | 155.359 | 609.883 | 1.97293 | - | GO:0005727 |  | hypothetical protein |
| DR_0686 | 80.9016 | 560.372 | 2.79215 | - | GO:0005727 |  | hypothetical protein |
| DR_0687 | 5.70531 | 17.5496 | 1.62106 | - | GO:0016021|GO:0005727 | | hypothetical protein |
| DR_0705 | 22.7026 | 57.383 | 1.33777 | COG2342G | GO:0004812|GO:0043169 | K01884:ko00970 Aminoacyl-tRNA biosynthesis | hypothetical protein |
| DR_0706 | 7.31556 | 19.79 | 1.43573 | COG3868S |  |  | hypothetical protein |
| DR_0707 | 12.6246 | 45.5369 | 1.8508 | - |  |  | hypothetical protein |
| DR_0708 | 103.941 | 335.165 | 1.6891 | - |  |  | hypothetical protein |
| DR_0709 | 22.351 | 104.99 | 2.23184 | COG0451MG | GO:0050662 |  | snoG protein |
| DR_0710 | 29.6952 | 78.388 | 1.4004 | COG1208MJ | GO:0016779 | K00966:ko00051 Fructose and mannose metabolism | mannose-1-phosphate guanyltransferase |
| DR_0713 | 39.0539 | 107.306 | 1.4582 | COG0438M | GO:0016740 |  | lipopolysaccharide glycosyltransferase, putative |
| DR_0736 | 6.83295 | 16.8072 | 1.2985 | - | GO:0006810 |  | hypothetical protein |
| DR_0737 | 16.5997 | 48.3574 | 1.54258 | - | GO:0006810 |  | hypothetical protein |
| DR_0746 | 9146.62 | 19450.4 | 1.08849 | - |  |  | hypothetical protein |
| DR_0748a | 21.4787 | 47.5594 | 1.14683 | COG1863P | GO:0016021 | K05569: | putative monovalent cation/H+ antiporter subunit E |
| DR_0784 | 12.8146 | 29.0506 | 1.18078 | COG1051F | GO:0016787 |  | MutT/nudix family protein |
| DR_0787 | 503.506 | 1093.54 | 1.11893 | COG2302S | GO:0003723 |  | hypothetical protein |
| DR_0816 | 6.47416 | 14.6816 | 1.18124 | COG1285S | GO:0016020|GO:0016021 | K07507: | Mg(2+) transport ATPase-like protein |
| DR_0827 | 19.7165 | 39.902 | 1.01706 | COG1011R | GO:0016787 | K07025: | CbbY/CbbZ/GpH/YieH family hydrolase |
| DR_0828 | 154.603 | 1544.49 | 3.32049 | COG2224C | GO:0019752 | K01637:ko00630 Glyoxylate and dicarboxylate metabolism | isocitrate lyase |
| DR_0829 | 23.2798 | 47.8438 | 1.03926 | COG2319R | GO:0043531 |  | WD repeat-containing protein |
| DR_0835 | 49.0961 | 127.796 | 1.38017 | - | GO:0006313 |  | hypothetical protein |
| DR_0850 | 0 | 4.77733 | 1.79769e+308 | - |  |  | hypothetical protein |
| DR_0859 | 509.17 | 1101.18 | 1.11283 | COG0684H | GO:0051252 | K02553: | ribonuclease activity regulator protein RraA |
| DR_0866 | 88.6699 | 359.306 | 2.01869 | COG0438M | GO:0009058|GO:0016740 | | lipopolysaccharide biosynthesis protein-like protein |
| DR_0883 | 19.541 | 55.8751 | 1.5157 | COG0651CP | GO:0055114 | K05568: | cation transport system protein, putative |
| DR_0890 | 101.055 | 246.336 | 1.28548 | COG1490J | GO:0019478 | K07560: | D-tyrosyl-tRNA(Tyr) deacylase |
| DR_0947 | 10.7295 | 24.8357 | 1.21083 | - | GO:0008152|GO:0016787 | | hypothetical protein |
| DR_0948 | 20.2686 | 44.8454 | 1.14571 | - |  |  | hypothetical protein |
| DR_0958 | 104.534 | 328.909 | 1.65371 | COG1173EP | GO:0016021 | K02034: | peptide ABC transporter permease |
| DR_0959 | 263.549 | 713.464 | 1.43677 | COG0601EP | GO:0016021 | K02033: | peptide ABC transporter permease |
| DR_0974 | 79.021 | 163.001 | 1.04457 | COG0563F |  |  | hypothetical protein |
| DR_0976 | 37.4811 | 78.2368 | 1.06168 | COG0216J | GO:0016149 | K02835: | peptide chain release factor 1 |
| DR_0993 | 13.7951 | 34.3879 | 1.31774 | - |  |  | hypothetical protein |
| DR_0996 | 0 | 14.6704 | 1.79769e+308 | - |  |  | hypothetical protein |
| DR_1002 | 6.83061 | 14.3227 | 1.06822 | COG2346R | GO:0020037 | K06886: | hypothetical protein |
| DR_1003 | 6.29314 | 19.0233 | 1.59592 | COG1309K | GO:0006355 |  | transcriptional regulator |
| DR_1010 | 1.16743 | 3.52367 | 1.59374 | - |  |  | hypothetical protein |
| DR_1014 | 39.8097 | 143.8 | 1.85287 | - | GO:0055114 |  | vanadium chloroperoxidase-like protein |
| DR_1015 | 25.5079 | 80.0389 | 1.64976 | - | GO:0055114 |  | hypothetical protein |
| DR_1021 | 426.737 | 934.298 | 1.13053 | COG0158G |  |  | hypothetical protein |
| DR_1030 | 1.10834 | 4.96771 | 2.16417 | COG1167KE | GO:0030170|GO:0080130 | | aminotransferase |
| DR_1043 | 24.7009 | 53.5715 | 1.1169 | - |  |  | hypothetical protein |
| DR_1045 | 5.92616 | 25.8067 | 2.12258 | - |  |  | hypothetical protein |
| DR_1048 | 14.5113 | 32.2353 | 1.15146 | - |  |  | hypothetical protein |
| DR_1049 | 9.4253 | 21.6791 | 1.20169 | COG4367S |  |  | hypothetical protein |
| DR_1050 | 29.8194 | 90.3812 | 1.59977 | - |  |  | hypothetical protein |
| DR_1053 | 7.97383 | 18.5999 | 1.22195 | COG1506E | GO:0016787 | K06889: | hydrolase |
| DR_1054 | 105.247 | 266.469 | 1.34018 | - |  |  | hypothetical protein |
| DR_1067 | 16122.2 | 44057 | 1.45032 | - | hypothetical protein | |  |
| DR_1079 | 46.2605 | 109.481 | 1.24283 | - |  |  | hypothetical protein |
| DR_1111 | 2.24875 | 8.29295 | 1.88276 | COG0789K | GO:0006355 |  | MerR family transcriptional regulator |
| DR_1112 | 1.69093 | 10.2965 | 2.60626 | COG3238S |  | K09936: | hypothetical protein |
| DR_1113 | 7.68246 | 23.9165 | 1.63836 | COG3238S |  | K09936: | hypothetical protein |
| DR_1121 | 92.78 | 253.754 | 1.45154 | - |  |  | hypothetical protein |
| DR_1140 | 8.07883 | 18.3439 | 1.18308 | - |  |  | hypothetical protein |
| DR_1150 | 79.9264 | 175.911 | 1.1381 | COG0621J | GO:0051539 | K06168: | (dimethylallyl)adenosine tRNA methylthiotransferase |
| DR_1155 | 203.801 | 598.022 | 1.55304 | COG2225C | GO:0016740|GO:0016746 | K01638:ko00620 Pyruvate metabolism | malate synthase |
| DR_1156 | 84.0269 | 302.241 | 1.84678 | COG1414K | GO:0006355 | K13641: | transcriptional regulator |
| DR_1157 | 14.2622 | 30.0491 | 1.07513 | COG2379G | GO:0055114|GO:0016740 | K00050:ko00260 Glycine, serine and threonine metabolism | hydroxypyruvate reductase, putative |
| DR_1176 | 126.435 | 394.714 | 1.64241 | - |  |  | hypothetical protein |
| DR_1180 | 447.024 | 896.566 | 1.00406 | - |  |  | hypothetical protein |
| DR_1193 | 20.8794 | 46.5835 | 1.15774 | - |  |  | hypothetical protein |
| DR_1194 | 23.8362 | 59.0125 | 1.30787 | - | GO:0016740 |  | hypothetical protein |
| DR_1205 | 85.9798 | 208.025 | 1.27469 | COG0274F | GO:0016829 | K01619:ko00030 Pentose phosphate pathway | deoxyribose-phosphate aldolase |
| DR_1208 | 0 | 5.14824 | 1.79769e+308 | COG1225O | GO:0055114 | K03564: | bacterioferritin comigratory protein |
| DR_1209 | 8.79009 | 32.1509 | 1.87091 | COG1225O | GO:0055114 | K03564: | bacterioferritin comigratory protein |
| DR_1220 | 29.6653 | 75.7651 | 1.35276 | COG1918P | GO:0005506 | K04758: | ferrous iron transport protein A |
| DR_1226 | 4.36082 | 15.9911 | 1.8746 | - | GO:0005975|GO:0016810 | | hypothetical protein |
| DR_1241 | 0 | 9.78053 | 1.79769e+308 | - |  |  | hypothetical protein |
| DR_1272 | 0 | 10.2548 | 1.79769e+308 | - |  |  | hypothetical protein |
| DR_1286 | 0 | 10.6229 | 1.79769e+308 | - |  |  | hypothetical protein |
| DR_1310 | 44.9724 | 98.3984 | 1.1296 | COG2137R | GO:0006282 | K03565: | recombination regulator RecX |
| DR_1332 | 2.40108 | 19.2344 | 3.00194 | - | GO:0016787|GO:0016798 | K01207:ko00520 Amino sugar and nucleotide sugar metabolism | hypothetical protein |
| DR_1346 | 34.815 | 91.2345 | 1.38987 | COG2355E | GO:0016805 | K01273: | peptidase |
| DR_1350 | 112.167 | 295.805 | 1.39899 | COG0075E | GO:0030170 | K00830:ko00250 Alanine, aspartate and glutamate metabolism | class V aminotransferase |
| DR_1358 | 15.2312 | 34.4602 | 1.1779 | COG1464P |  | K02073:ko02010 ABC transporters | outer membrane protein |
| DR_1389 | 31.4097 | 63.7994 | 1.02233 | COG1040R | GO:0016740|GO:0016757 | | competence protein ComF |
| DR_1450 | 30.2741 | 86.5 | 1.51461 | COG1380R | GO:0016021 | K06518: | hypothetical protein |
| DR_1451 | 54.2103 | 135.253 | 1.31902 | COG0775F | GO:0019509 | K01243:ko00270 Cysteine and methionine metabolism | 5`-methylthioadenosine/S-adenosylhomocysteine nuclosidase |
| DR_1464 | 40.4344 | 129.675 | 1.68125 | - |  |  | hypothetical protein |
| DR_1466 | 15.1437 | 45.3847 | 1.58349 | - |  |  | hypothetical protein |
| DR_1484 | 41.4277 | 118.409 | 1.51512 | - |  |  | hypothetical protein |
| DR_1527 | 2.36898 | 13.1311 | 2.47064 | - |  |  | hypothetical protein |
| DR_1558 | 3.71859 | 8.32332 | 1.1624 | COG2197TK | GO:0043565 |  | DNA-binding response regulator |
| DR_1566 | 8.67993 | 21.7223 | 1.32342 | - |  |  | hypothetical protein |
| DR_1580 | 5.55469 | 36.1694 | 2.70299 | - | GO:0016702 |  | hypothetical protein |
| DR_1594 | 28.6333 | 99.5834 | 1.79821 | - |  |  | hypothetical protein |
| DR_1614 | 49.2255 | 109.544 | 1.15403 | COG0066E | GO:0016829|GO:0009316 | K01704:ko00290 Valine, leucine and isoleucine biosynthesis | 3-isopropylmalate dehydratase small subunit |
| DR_1616 | 584.06 | 1265.47 | 1.11548 | COG4371S |  |  | hypothetical protein |
| DR_1626 | 190.643 | 452.109 | 1.2458 | COG0115EH | GO:0052656 | K00826:ko00280 Valine, leucine and isoleucine degradation | branched-chain amino acid aminotransferase |
| DR_1642 | 25.3639 | 54.5713 | 1.10537 | COG2318S | GO:0016787 |  | hypothetical protein |
| DR_1695 | 39.3438 | 110.503 | 1.48988 | COG3324R | GO:0016829|GO:0016702 | K01759:ko00620 Pyruvate metabolism | lactoylglutathione lyase-like protein |
| DR_1697 | 50.9721 | 121.947 | 1.25848 | - |  |  | hypothetical protein |
| DR_1708 | 276.799 | 725.99 | 1.39111 | COG4719S | GO:0005727 |  | hypothetical protein |
| DR_1744 | 11.888 | 48.1744 | 2.01876 | - | GO:0016757 |  | hypothetical protein |
| DR_1751 | 6.4425 | 16.1079 | 1.32207 | COG1573L |  | K02334: | DNA polymerase-like protein |
| DR_1758 | 22.0978 | 46.5496 | 1.07487 | COG0019E | GO:0030170|GO:0016831 | K01586:ko00300 Lysine biosynthesis | diaminopimelate decarboxylase |
| DR_1772 | 77.2946 | 205.834 | 1.41304 | - |  |  | hypothetical protein |
| DR_1777 | 6.51374 | 19.3647 | 1.57187 | - |  |  | hypothetical protein |
| DR_1791 | 2.77717 | 6.37014 | 1.19771 | COG0147EH | GO:0016833 | K01657:ko00400 Phenylalanine, tyrosine and tryptophan biosynthesi | anthranilate synthase component I |
| DR_1794 | 5.21627 | 16.0018 | 1.61715 | COG1477H | GO:0042597 | K03734: | nosX protein |
| DR_1795 | 12.4864 | 25.3436 | 1.02127 | - |  |  | hypothetical protein |
| DR_1805 | 38.189 | 77.0941 | 1.01346 | - | GO:0043169 |  | hypothetical protein |
| DR_1806 | 9.62866 | 34.0158 | 1.8208 | COG1669R | GO:0016779 | K07075: | hypothetical protein |
| DR_1810 | 48.0473 | 214.694 | 2.15976 | - |  |  | hypothetical protein |
| DR_1814 | 9.5157 | 37.6066 | 1.98261 | COG1514J | GO:0016070 |  | hypothetical protein |
| DR_1828 | 10.4926 | 43.3736 | 2.04745 | - |  |  | hypothetical protein |
| DR_1850 | 52.2615 | 124.698 | 1.25462 | - |  |  | hypothetical protein |
| DR_1853 | 1.2514 | 3.0898 | 1.30397 | - |  |  | hypothetical protein |
| DR_1862 | 1.5473 | 3.10653 | 1.00555 | COG0009J |  | K07566: | SUA5-like protein |
| DR_1872 | 41.222 | 92.4435 | 1.16515 | - | GO:0006355 |  | hypothetical protein |
| DR_1889 | 30.305 | 76.136 | 1.32902 | COG2389R | GO:0016787 |  | hypothetical protein |
| DR_1920 | 9.57072 | 22.0291 | 1.20271 | - |  |  | hypothetical protein |
| DR_1927 | 140.865 | 15000.2 | 6.73452 | COG1943L | GO:0006313 | K07491: | transposase |
| DR_1949 | 4.03288 | 18.0803 | 2.16454 | COG0164L | GO:0090305 | K03470:ko03030 DNA replication | ribonuclease HII |
| DR_1974 | 517.151 | 1365.77 | 1.40106 | COG0466O | GO:0017111 | K01338:ko04112 Cell cycle - Caulobacter | ATP-dependent protease LA |
| DR_2003 | 44.3624 | 136.014 | 1.61634 | - |  |  | hypothetical protein |
| DR_2015 | 44.9413 | 97.1084 | 1.11155 | COG1233Q | GO:0055114 |  | oxidoreductase, FAD-binding |
| DR_2039 | 14.4474 | 31.8686 | 1.14133 | - |  |  | hypothetical protein |
| DR_2053 | 1.23184 | 48.3069 | 5.29334 | COG0704P |  |  | phosphate transport system regulatory protein PhoU |
| DR_2054 | 52.0151 | 136.611 | 1.39307 | - | GO:0008270 |  | hypothetical protein |
| DR_2056 | 6.98443 | 15.8774 | 1.18476 | COG3187O |  |  | hypothetical protein |
| DR_2064 | 18.2554 | 37.5181 | 1.03926 | - |  |  | hypothetical protein |
| DR_2114 | 229.204 | 518.695 | 1.17825 | COG1841J | GO:0030529 | K02907:ko03010 Ribosome | 50S ribosomal protein L30 |
| DR_2152 | 15301.6 | 95175.4 | 2.6369 | COG0230J | GO:0030529 | K02914:ko03010 Ribosome | 50S ribosomal protein L34 |
| DR_2171 | 2.97346 | 5.96587 | 1.00459 | - |  |  | hypothetical protein |
| DR_2228 | 148.501 | 330.663 | 1.15489 | - |  |  | hypothetical protein |
| DR_2231 | 37.4933 | 76.0592 | 1.02049 | COG4696S | GO:0046872 |  | hypothetical protein |
| DR_2234 | 60.2611 | 141.899 | 1.23556 | COG1597IR | GO:0007205|GO:0016310 | K07029: | hypothetical protein |
| DR_2237 | 16.4673 | 39.1484 | 1.24935 | - |  |  | hypothetical protein |
| DR_2247 | 91.6583 | 233.404 | 1.34849 | COG3427S |  | K09386: | hypothetical protein |
| DR_2259 | 7.43735 | 24.9819 | 1.74802 | COG1476K | GO:0043565 |  | transcriptional regulator |
| DR_2281 | 6.69978 | 16.3143 | 1.28395 | COG0571K | GO:0016075|GO:0005622 | | hypothetical protein |
| DR_2283 | 22.3848 | 89.2705 | 1.99566 | COG1108P | GO:0042626 | K09819: | manganese ABC transporter permease |
| DR_2284 | 60.7718 | 317.946 | 2.38731 | COG1121P | GO:0017111 | K09820: |K11607:ko02010 ABC transporters | manganese ABC transporter ATP-binding protein |
| DR_2292 | 3.30432 | 8.7585 | 1.40633 | - |  |  | hypothetical protein |
| DR_2313 | 41.6196 | 177.308 | 2.09092 | - |  |  | hypothetical protein |
| DR_2363 | 264.594 | 580.294 | 1.133 | COG0589T | GO:0006950 |  | hypothetical protein |
| DR_2374 | 18.3773 | 60.8005 | 1.72616 | COG0209F | GO:0055114 | K00525:ko00230 Purine metabolism | ribonucleoside-diphosphate reductase-like protein |
| DR_2375 | 11.3598 | 25.8232 | 1.18473 | COG0782K | GO:0032784 | K03624: | transcription elongation factor |
| DR_2424 | 6.89391 | 19.8941 | 1.52895 | COG0675L |  | K07496: | putative transposase |
| DR_2458 | 8.62754 | 45.066 | 2.38502 | - |  |  | hypothetical protein |
| DR_2460 | 22.228 | 61.4608 | 1.46729 | COG0727R |  | K06940: | hypothetical protein |
| DR_2463 | 39.4526 | 92.859 | 1.23492 | COG0598P | GO:0055085 | K03284: | transport protein |
| DR_2519 | 28.331 | 73.2208 | 1.36988 | COG0789K | GO:0006355 |  | MerR family transcriptional regulator |
| DR_2520 | 5.22615 | 58.6814 | 3.48908 | - |  |  | hypothetical protein |
| DR_2522 | 16.3175 | 101.164 | 2.63221 | COG2267I | GO:0016787 |  | lipase |
| DR_2523 | 35.0086 | 487.163 | 3.79862 | COG0803P | GO:0046872 | K09818: |K02077: | adhesin B |
| DR_2537 | 1.84856 | 12.6605 | 2.77586 | COG2717S | GO:0055114|GO:0016021 | | putative sulfite oxidase subunit YedZ |
| DR_2561 | 779.799 | 3762.7 | 2.2706 | COG4895S |  |  | hypothetical protein |
| DR_2562 | 890.874 | 1812.63 | 1.02479 | COG2226H | GO:0032259 | K00568:ko00130 Ubiquinone and other terpenoid-quinone biosynthesi | 3-demethylubiquinone-9 3-methyltransferase |
| DR_2566 | 0.806968 | 2.94452 | 1.86745 | COG2852S | GO:0016853 |  | hypothetical protein |
| DR_2585 | 8.56857 | 20.6358 | 1.26803 | COG1853R | GO:0055114 |  | hypothetical protein |
| DR_2591 | 53.6096 | 131.331 | 1.29265 | COG3411C | GO:0055114 |  | hypothetical protein |
| DR_2593 | 127.878 | 282.012 | 1.14099 | - |  |  | hypothetical protein |
| DR_2628 | 13.5239 | 31.9573 | 1.24063 | - |  |  | hypothetical protein |
| DR_A0017 | 41.9623 | 103.058 | 1.29629 | COG2050Q |  |  | hypothetical protein |
| DR_A0051 | 7.62396 | 16.9039 | 1.14874 | COG3230P | GO:0055114 |  | Heme oxygenase |
| DR_A0072 | 10.8024 | 112.164 | 3.37619 | COG0695O | GO:0045454 |  | Glutaredoxin |
| DR_A0076 | 3.51278 | 11.0799 | 1.65726 | COG2842R | GO:0017111|GO:0043565 | K07132: | hypothetical protein |
| DR_A0085 | 4.94421 | 20.6632 | 2.06325 | - |  |  | hypothetical protein |
| DR_A0088 | 10.6106 | 26.2647 | 1.30763 | - |  |  | hypothetical protein |
| DR_A0090 | 4.08305 | 8.75305 | 1.10014 | - |  |  | hypothetical protein |
| DR_A0092 | 2.06982 | 21.023 | 3.34439 | - |  |  | hypothetical protein |
| DR_A0100 | 0 | 4.77299 | 1.79769e+308 | - |  |  | hypothetical protein |
| DR_A0102 | 12.9736 | 35.0328 | 1.43313 | - |  |  | hypothetical protein |
| DR_A0105 | 1.43072 | 13.1965 | 3.20534 | - |  |  | hypothetical protein |
| DR_A0116 | 1.53032 | 8.36429 | 2.45041 | - |  |  | hypothetical protein |
| DR_A0165 | 24.9877 | 66.8219 | 1.4191 | - |  |  | hypothetical protein |
| DR_A0168 | 2.98494 | 8.70687 | 1.54445 | COG0555O | GO:0016021 | K02018:ko02010 ABC transporters | molybdenum ABC transporter permease |
| DR_A0174 | 1.99753 | 5.27361 | 1.40057 | COG0352H | GO:0046872 | K00788:ko00730 Thiamine metabolism | thiamin-phosphate pyrophosphorylase |
| DR_A0191 | 2.11146 | 9.69782 | 2.19942 | COG2971G | GO:0016310 |  | hypothetical protein |
| DR_A0219 | 39.2791 | 102.458 | 1.3832 | - |  |  | hypothetical protein |
| DR_A0220 | 57.0717 | 114.728 | 1.00737 | COG1012C | GO:1901023 | K00151:ko00350 Tyrosine metabolism | aldehyde dehydrogenase |
| DR_A0234 | 8806.63 | 18439.4 | 1.06613 | - |  |  | hypothetical protein |
| DR_A0239 | 7.98669 | 16.6577 | 1.06052 | COG0368H | GO:0016740 | K02233:ko00860 Porphyrin and chlorophyll metabolism | cobalamin synthase |
| DR_A0248 | 14.7923 | 30.5176 | 1.04479 | COG1846K | GO:0006355 |  | MarR family transcriptional regulator |
| DR_A0254 | 15.0673 | 58.165 | 1.94873 | - | GO:0006313 | K07495: | resolvase |
| DR_A0268 | 4.18245 | 9.66523 | 1.20846 | - | GO:0016810 | K01486:ko00230 Purine metabolism |K02008:ko02010 ABC transporters | adenine deaminase-like protein |
| DR_A0276 | 223.147 | 522.457 | 1.22732 | COG0281C | GO:0055114 | K00027:ko00620 Pyruvate metabolism | malate dehydrogenase |
| DR_A0277 | 606.775 | 1350.11 | 1.15385 | COG2225C | GO:0016740|GO:0016746 | K01638:ko00620 Pyruvate metabolism | malate synthase |
| DR_A0278 | 134.876 | 286.846 | 1.08865 | - |  |  | hypothetical protein |
| DR_A0291 | 31.1837 | 63.1903 | 1.01891 | COG3836G | GO:0046872 | K02510:ko00350 Tyrosine metabolism | 2,4-dihydroxyhept-2-ene-1,7-dioic acid aldolase |
| DR_A0292 | 51.6459 | 143.3 | 1.47231 | - |  |  | hypothetical protein |
| DR_A0300 | 4.44115 | 9.54937 | 1.10447 | - |  |  | hypothetical protein |
| DR_A0301 | 12.6678 | 34.4903 | 1.44502 | COG1858P | GO:0055114 | K00428: | methylamine utilization protein |
| DR_A0303 | 0 | 5.25325 | 1.79769e+308 | - |  |  | hypothetical protein |
| DR_A0304 | 36.2963 | 92.2925 | 1.34639 | COG0491R | GO:0016787 | K01069:ko00620 Pyruvate metabolism | hypothetical protein |
| DR_A0309 | 26.3039 | 57.5746 | 1.13015 | COG0318IQ | GO:0016874|GO:0008152 | K01897:ko00071 Fatty acid metabolism |K00666: | acyl-CoA synthetase |
| DR_A0323 | 226.58 | 474.144 | 1.06531 | COG4674R | GO:0017111|GO:0016887 | K01995:ko02010 ABC transporters | urea/short-chain amide ABC transporter ATP-binding protein |
| DR_A0361 | 12.5869 | 28.4194 | 1.17495 | COG0598P | GO:0055085 | K03284: | cation transporter putative |
| DR_A0363 | 13.3701 | 41.9427 | 1.64941 | COG1233Q | GO:0055114 |  | P49 secreted protein |
| DR_B0054 | 243.703 | 557.962 | 1.19504 | - |  |  | hypothetical protein |
| DR_B0055 | 0 | 15.6651 | 1.79769e+308 | - | GO:0006313 | K07495: | transposase, pseudogene |
| DR_B0056 | 55.4353 | 159.006 | 1.5202 |  |  |  |  |
| DR_B0084 | 0 | 28.1437 | 1.79769e+308 | - |  |  | hypothetical protein |
| DR_B0085 | 0 | 9.78053 | 1.79769e+308 | - | GO:0051365 |  | hypothetical protein |
| DR_B0088 | 1.44413 | 3.7988 | 1.39534 | COG2205T | GO:0023014 | K07646:ko02020 Two-component system | KdpD-related protein |
| DR_B0100 | 14.0092 | 28.8941 | 1.0444 | - | GO:0016874 |  | hypothetical protein |
| DR_B0105 | 1859.77 | 7018.82 | 1.9161 | - |  |  | hypothetical protein |
| DR_B0107 | 1483.11 | 4055.86 | 1.45138 | COG1780F | GO:0010181 | K03647: | ribonucleotide reductase |

Table S2: The repressed genes showed in Mt-0865. All genes are sorted by gene id.

| **Synonym** | **R1** | **Mt0865** | **fold_change** | **COG** | **GO** | **KO** | **Product** |
| --- | --- | --- | --- | --- | --- | --- | --- |
| DR_0014 | 88.7007 | 31.7177 | -1.48366 | COG0357M | GO:0070476 | K03501: | 16S rRNA methyltransferase GidB |
| DR_0034 | 31.1954 | 15.5668 | -1.00286 | - |  |  | hypothetical protein |
| DR_0057 | 68.639 | 27.4078 | -1.32444 | COG0695O |  |  | hypothetical protein |
| DR_0059 | 59.3851 | 19.1171 | -1.63524 | - |  |  | hypothetical protein |
| DR_0082 | 299.661 | 132.134 | -1.18133 | - |  |  | hypothetical protein |
| DR_0090 | 5.60817 | 1.22807 | -2.19113 | - | GO:0016746 |  | hypothetical protein |
| DR_0091 | 16.0603 | 6.69254 | -1.26288 | COG2324S | GO:0016872 | K08977: | hypothetical protein |
| DR_0122 | 22.2787 | 9.59081 | -1.21594 | - | GO:0046872|GO:0008152 | | hypothetical protein |
| DR_0130 | 15.8524 | 7.38417 | -1.10219 | COG0635H | GO:0055114 | K02495:ko00860 Porphyrin and chlorophyll metabolism | coproporphyrinogen III oxidase |
| DR_0139 | 192.869 | 66.5164 | -1.53584 | COG2262R | GO:0006184|GO:0016787 | K03665: | GTP-binding protein HflX |
| DR_0160 | 70.6717 | 29.1901 | -1.27565 | - |  |  | hypothetical protein |
| DR_0161 | 21.2561 | 7.55596 | -1.49219 | COG2327S | GO:0016740 |  | hypothetical protein |
| DR_0205 | 10.4848 | 2.86651 | -1.87094 | COG4586R | GO:0017111 | K01990: | ABC transporter ATP-binding protein |
| DR_0242 | 13.1738 | 0 | -1.79769e+308 | COG3502S | GO:0016740 |  | hypothetical protein |
| DR_0244 | 50.0636 | 18.467 | -1.43881 | - | GO:0008080|GO:0016740 | | hypothetical protein |
| DR_0291 | 710.142 | 265.723 | -1.41818 | COG1579R |  | K07164: | hypothetical protein |
| DR_0298 | 33.347 | 15.1037 | -1.14266 | COG1428F | GO:0016773 | K00924: | deoxyguanosine kinase/deoxyadenosine kinase subunit |
| DR_0336 | 118.709 | 52.6025 | -1.17423 | COG0318IQ | GO:0016874 | K00666: | fatty-acid--CoA ligase |
| DR_0342 | 57.099 | 20.8359 | -1.4544 | COG0723C | GO:0055114 |  | cytochrome complex iron-sulfur subunit |
| DR_0344 | 45.3106 | 21.3061 | -1.08858 | COG3088O |  | K02200: | cytochrome c-type biogenesis protein CcmH |
| DR_0346 | 59.0954 | 27.6801 | -1.0942 | COG1138O | GO:0017004 | K02198: | cytochrome c-type biogenesis protein CcmF |
| DR_0347 | 288.087 | 116.799 | -1.30247 | COG2332O | GO:0046872 | K02197: | cytochrome c-type biogenesis protein CcmE |
| DR_0348 | 84.2236 | 30.5999 | -1.4607 | COG0755O | GO:0017004 | K02195:ko02010 ABC transporters | cytochrome c-type biogenesis heme exporter protein C |
| DR_0352 | 27.8008 | 13.1285 | -1.08243 | - | GO:0090305 | K12573:ko03018 RNA degradation | hypothetical protein |
| DR_0354 | 8.27768 | 2.36469 | -1.80757 | COG0708L | GO:0090305 | K01142:ko03410 Base excision repair | exodeoxyribonuclease III |
| DR_0359 | 15.8222 | 4.28679 | -1.88398 | - |  |  | hypothetical protein |
| DR_0378 | 84.1434 | 41.4492 | -1.02151 | COG1309K | GO:0006355 |  | TetR family transcriptional regulator |
| DR_0393 | 57.9036 | 21.4374 | -1.43352 | COG0480J | GO:0006414|GO:0006184 | K02355: | elongation factor G |
| DR_0395 | 22.5334 | 10.8485 | -1.05457 | COG2814G | GO:0055085 |  | hypothetical protein |
| DR_0426 | 20.3437 | 9.54584 | -1.09164 | COG0118E | GO:0016763 | K02501:ko00340 Histidine metabolism | imidazole glycerol phosphate synthase subunit HisH |
| DR_0441 | 287.678 | 99.021 | -1.53865 | COG2339S | GO:0008233 |  | hypothetical protein |
| DR_0448 | 124.715 | 11.5974 | -3.42675 | - | GO:0043565|GO:0003677 | | hypothetical protein |
| DR_0455 | 14.5845 | 4.72938 | -1.62471 | COG3570V | GO:0019748 |  | streptomycin 3-kinase |
| DR_0457 | 106.148 | 38.5594 | -1.46093 | COG0848U | GO:0016021 | K03559: | biopolymer transport protein |
| DR_0461 | 16.0872 | 6.26837 | -1.35975 | COG1521K | GO:0046872 | K03525:ko00770 Pantothenate and CoA biosynthesis | pantothenate kinase |
| DR_0465 | 3.26241 | 1.5791 | -1.04683 | COG2340S |  |  | hypothetical protein |
| DR_0477 | 128.072 | 55.5238 | -1.20578 | COG4147R | GO:0055085 | K14393: | sodium:solute symporter protein |
| DR_0483 | 16.8234 | 7.99144 | -1.07394 | COG3012S |  | K09858: | hypothetical protein |
| DR_0505 | 87.7792 | 17.3927 | -2.3354 | COG0737F | GO:0016787 | K01081:ko00230 Purine metabolism | 5`-nucleotidase family protein |
| DR_0508 | 59.9675 | 29.0146 | -1.0474 | COG1715V | GO:0090305 | K07448: | mrr restriction system protein |
| DR_0509 | 20.3341 | 9.68157 | -1.07059 | - |  |  | hypothetical protein |
| DR_0510 | 49.9565 | 15.1152 | -1.72467 | - | GO:0007165 |  | sensory box protein |
| DR_0519 | 4.24832 | 0 | -1.79769e+308 | - |  |  | hypothetical protein |
| DR_0520 | 6.68586 | 1.07139 | -2.64163 | - |  |  | hypothetical protein |
| DR_0533 | 74.0454 | 35.6489 | -1.05455 | - |  |  | hypothetical protein |
| DR_0539 | 24.4153 | 2.18937 | -3.47919 | - |  |  | hypothetical protein |
| DR_0542 | 19.6554 | 7.04281 | -1.4807 | COG0584C | GO:0008889 | K01126:ko00564 Glycerophospholipid metabolism | glycerophosphoryl diester phosphodiesterase |
| DR_0545 | 45.7361 | 15.4617 | -1.56463 | - |  |  | hypothetical protein |
| DR_0548 | 302.068 | 102.914 | -1.55343 | - |  | K02650:ko02020 Two-component system | pilin, type IV |
| DR_0605 | 96.5878 | 20.8738 | -2.21015 | COG2199T | GO:0035556 |  | GGDEF family protein |
| DR_0622 | 10.0497 | 0 | -1.79769e+308 | - |  |  | hypothetical protein |
| DR_0635 | 134.299 | 66.5304 | -1.01336 | COG0205G | GO:0046872 | K00850:ko00010 Glycolysis / Gluconeogenesis | 6-phosphofructokinase |
| DR_0655 | 45.6205 | 0 | -1.79769e+308 | - |  | K07093: | hypothetical protein |
| DR_0668 | 71.8422 | 30.6303 | -1.22987 | COG0458EF | GO:0046872 | K01955:ko00240 Pyrimidine metabolism | carbamoyl phosphate synthase large subunit |
| DR_0675 | 163.322 | 71.2748 | -1.19625 | COG0456R | GO:0008080|GO:0016740 | | hypothetical protein |
| DR_0720 | 14.1276 | 2.72738 | -2.37293 | COG1309K | GO:0006355 |  | TetR family transcriptional regulator |
| DR_0733 | 51.0136 | 19.3955 | -1.39516 | COG0139E | GO:0016787 | K11755:ko00340 Histidine metabolism | bifunctional phosphoribosyl-AMP cyclohydrolase/phosphoribosyl-ATP pyrophosphatase protein |
| DR_0742 | 102.902 | 48.691 | -1.07954 | COG1438K | GO:0051259 | K03402: | arginine repressor/activator |
| DR_0780 | 23.5866 | 10.1759 | -1.21281 | - |  |  | hypothetical protein |
| DR_0798 | 8.93224 | 2.87689 | -1.63451 | COG1670J | GO:0016740 |  | acetyltransferase |
| DR_0807 | 58.3802 | 27.9462 | -1.06283 | COG0682M | GO:0042158 | K13292: | prolipoprotein diacylglyceryl transferase |
| DR_0811 | 49.2192 | 13.8783 | -1.8264 | COG3169S |  | K09922: | hypothetical protein |
| DR_0823 | 211.493 | 102.585 | -1.0438 | COG1940KG | GO:0016740|GO:0047330 | K00886:ko00010 Glycolysis / Gluconeogenesis | ROK family protein |
| DR_0851 | 26.7781 | 12.9153 | -1.05197 | COG0382H | GO:0016740 | K03179:ko00130 Ubiquinone and other terpenoid-quinone biosynthesi | 4-hydroxybenzoate octaprenyltransferase |
| DR_0875 | 38.9743 | 16.3472 | -1.25348 | COG0308E | GO:0008270 |  | zinc metalloprotease |
| DR_0885 | 44.6006 | 21.0254 | -1.08493 | COG2212P | GO:0034220 | K05570: |K05563: | cation transport system protein |
| DR_0910 | 29.9371 | 12.5136 | -1.25844 | COG1388M | GO:0016998 |  | cell wall protein |
| DR_0943 | 11.3578 | 2.38867 | -2.2494 | COG1547S |  | K09763: | hypothetical protein |
| DR_0994 | 57.6354 | 4.12089 | -3.80593 | - |  |  | hypothetical protein |
| DR_0995 | 104.505 | 36.9712 | -1.49909 | COG2018R |  | K07131: | hypothetical protein |
| DR_1013 | 34.7664 | 17.1422 | -1.02014 | - |  | K01992: |K09686:ko02010 ABC transporters | hypothetical protein |
| DR_1019 | 866.645 | 255.273 | -1.7634 | COG0578C | GO:0055114 | K00111:ko00564 Glycerophospholipid metabolism | glycerol-3-phosphate dehydrogenase |
| DR_1052 | 15.473 | 5.59176 | -1.46838 | COG0421E | GO:0008152 |  | hypothetical protein |
| DR_1071 | 75.7184 | 23.8867 | -1.66444 | COG2350S |  |  | hypothetical protein |
| DR_1076 | 49.4803 | 20.3966 | -1.27853 | COG0707M | GO:0030259 |  | cell wall synthesis protein |
| DR_1081 | 33.4565 | 12.8198 | -1.38391 | COG1309K | GO:0006355 |  | TetR family transcriptional regulator |
| DR_1089 | 47.4286 | 21.138 | -1.16592 | COG1195L | GO:0017111|GO:0009432 | K03629:ko03440 Homologous recombination | recombination protein F |
| DR_1095 | 9.88843 | 0 | -1.79769e+308 | - |  |  | hypothetical protein |
| DR_1100 | 24.1866 | 7.02865 | -1.78289 | COG1054R | GO:0016740 | K07146: | hypothetical protein |
| DR_1107 | 187.688 | 85.601 | -1.13264 | - |  |  | hypothetical protein |
| DR_1108 | 80.4645 | 31.6218 | -1.34743 | - |  |  | hypothetical protein |
| DR_1109 | 251.839 | 104.574 | -1.26798 | COG0540F | GO:0016743 | K00609:ko00240 Pyrimidine metabolism | aspartate carbamoyltransferase catalytic subunit |
| DR_1110 | 196.666 | 83.495 | -1.23599 | COG2065F | GO:0016757 | K02825:ko00240 Pyrimidine metabolism | bifunctional pyrimidine regulatory protein PyrR uracil phosphoribosyltransferase |
| DR_1114 | 1435.74 | 591.034 | -1.28048 | COG0071O | GO:0006950 | K13993:ko04141 Protein processing in endoplasmic reticulum | HSP20 family protein |
| DR_1129 | 15.9102 | 3.69745 | -2.10535 | - |  |  | hypothetical protein |
| DR_1133 | 186.622 | 81.83 | -1.18942 | COG0407H | GO:0016831 | K01599:ko00860 Porphyrin and chlorophyll metabolism | uroporphyrinogen decarboxylase |
| DR_1136 | 118.449 | 53.9832 | -1.13369 | - |  |  | hypothetical protein |
| DR_1163 | 433.569 | 178.759 | -1.27825 | COG2805NU | GO:0017111 | K02669: | twitching motility protein |
| DR_1182 | 23.72 | 10.223 | -1.21428 | COG1247M | GO:0016740|GO:0008080 | K03823:ko00440 Phosphonate and phosphinate metabolism | phosphinothricin acetyltransferase |
| DR_1189 | 2330.25 | 1060.48 | -1.13576 | COG1884I | GO:0031419 | K01848:ko00280 Valine, leucine and isoleucine degradation | methylmalonyl-CoA mutase subunit alpha, chain A |
| DR_1190 | 166.772 | 74.5189 | -1.1622 | COG3548S |  |  | hypothetical protein |
| DR_1222 | 18.8638 | 7.36845 | -1.35619 | COG0726G | GO:0016810 |  | polysaccharide deacetylase |
| DR_1236 | 196.533 | 61.1067 | -1.68537 | COG0053P | GO:0055085 |  | hypothetical protein |
| DR_1247 | 2168.27 | 969.727 | -1.16089 | COG0045C | GO:0046872 | K01903:ko00020 Citrate cycle (TCA cycle) | succinyl-CoA synthetase subunit beta |
| DR_1267 | 4.22732 | 2.05929 | -1.03759 | - |  |  | hypothetical protein |
| DR_1270 | 83.7783 | 36.2775 | -1.2075 | COG0017J | GO:0016874 | K01893:ko00970 Aminoacyl-tRNA biosynthesis | asparaginyl-tRNA synthetase |
| DR_1280 | 164.385 | 78.8483 | -1.05993 | - |  |  | hypothetical protein |
| DR_1284 | 12.062 | 2.69339 | -2.16298 | - | GO:0006313|GO:0003677 | K07495: | hypothetical protein |
| DR_1299 | 47.1109 | 8.40395 | -2.48692 | - |  |  | hypothetical protein |
| DR_1302 | 49.877 | 12.9052 | -1.95043 | COG3842E | GO:0043190 | K11072:ko02010 ABC transporters | spermidine/putrescine ABC transporter ATP-binding protein |
| DR_1303 | 27.7684 | 10.4496 | -1.41 | COG1176E | GO:0016021 | K11071:ko02010 ABC transporters | spermidine/putrescine ABC transporter permease |
| DR_1422 | 689.762 | 341.085 | -1.01597 | - |  |  | hypothetical protein |
| DR_1444 | 101.148 | 33.7771 | -1.58235 | COG3705E | GO:0016874|GO:0016757 | K02502:ko00340 Histidine metabolism | ATP phosphoribosyltransferase regulatory subunit |
| DR_1445 | 80.9564 | 20.0644 | -2.01251 | COG0040E | GO:0016757 | K00765:ko00340 Histidine metabolism | ATP phosphoribosyltransferase catalytic subunit |
| DR_1446 | 237.892 | 67.4326 | -1.81879 | - |  |  | hypothetical protein |
| DR_1455 | 565.348 | 150.587 | -1.90854 | - | GO:0003677 |  | hypothetical protein |
| DR_1471 | 8.28173 | 3.28038 | -1.33607 | COG1196D | GO:0051276 | K03529: | Smc1/Cut3/Cut14 family protein |
| DR_1481 | 80.625 | 32.2343 | -1.32263 | COG3253S |  | K09162: | hypothetical protein |
| DR_1492 | 42.6928 | 20.9471 | -1.02724 | COG1007C | GO:0055114 | K00343:ko00190 Oxidative phosphorylation | NADH dehydrogenase I subunit N |
| DR_1493 | 42.4098 | 13.2178 | -1.68191 | COG1008C | GO:0055114 | K00342:ko00190 Oxidative phosphorylation | NADH dehydrogenase I subunit M |
| DR_1494 | 74.4489 | 25.3451 | -1.55454 | COG1009CP | GO:0055114 | K00341:ko00190 Oxidative phosphorylation | NADH dehydrogenase I subunit L |
| DR_1497 | 78.3992 | 13.4637 | -2.54177 | COG1143C | GO:0055114 | K00338:ko00190 Oxidative phosphorylation | NADH dehydrogenase I subunit I |
| DR_1498 | 57.5405 | 11.8731 | -2.27688 | COG1005C | GO:0055114 | K00337:ko00190 Oxidative phosphorylation | NADH dehydrogenase I subunit H |
| DR_1499 | 84.9471 | 28.558 | -1.57267 | COG1034C | GO:0055114 | K00336:ko00190 Oxidative phosphorylation | NADH dehydrogenase I subunit G |
| DR_1500 | 108.412 | 27.1361 | -1.99824 | COG1894C | GO:0055114 | K00335:ko00190 Oxidative phosphorylation | NADH dehydrogenase I subunit F |
| DR_1501 | 85.1541 | 25.1728 | -1.75821 | COG1905C | GO:0055114 | K00334:ko00190 Oxidative phosphorylation | NADH dehydrogenase I subunit E |
| DR_1502 | 148.741 | 34.0345 | -2.12774 | - |  |  | hypothetical protein |
| DR_1503 | 198.266 | 33.0388 | -2.58521 | COG0649C | GO:0055114 | K00333:ko00190 Oxidative phosphorylation | NADH dehydrogenase I subunit D |
| DR_1504 | 109.024 | 21.9023 | -2.31549 | COG0852C | GO:0055114 | K00332:ko00190 Oxidative phosphorylation | NADH dehydrogenase I subunit C |
| DR_1505 | 152.247 | 43.7563 | -1.79885 | COG0377C | GO:0055114 | K00331:ko00190 Oxidative phosphorylation | NADH dehydrogenase subunit B |
| DR_1506 | 201.927 | 74.2607 | -1.44316 | COG0838C | GO:0055114 | K00330:ko00190 Oxidative phosphorylation | NADH dehydrogenase I subunit A |
| DR_1547 | 6.72296 | 0 | -1.79769e+308 | - |  |  | hypothetical protein |
| DR_1548 | 32.6579 | 11.9158 | -1.45456 | COG2340S |  |  | hypothetical protein |
| DR_1550 | 20.7073 | 7.54204 | -1.45711 | COG2884D | GO:0051301 | K09812:ko02010 ABC transporters | ftsE protein |
| DR_1555 | 34.4102 | 16.7972 | -1.03461 | COG0438M | GO:0009058|GO:0016740 | | hypothetical protein |
| DR_1561 | 52.4928 | 22.7519 | -1.20613 | COG0381M | GO:0016853 | K01791:ko00520 Amino sugar and nucleotide sugar metabolism | UDP-N-acetylglucosamine 2-epimerase |
| DR_1576 | 450.219 | 36.5549 | -3.62249 | - |  |  | hypothetical protein |
| DR_1619 | 250.957 | 115.386 | -1.12098 | COG2720V |  |  | hypothetical protein |
| DR_1628 | 42.6262 | 11.8564 | -1.84608 | COG0789K | GO:0045892 |  | transcriptional activator TipA |
| DR_1640 | 111.031 | 0 | -1.79769e+308 | - |  |  | hypothetical protein |
| DR_1657 | 9.95637 | 3.72293 | -1.41918 | - |  |  | hypothetical protein |
| DR_1667 | 7.97915 | 2.11548 | -1.91525 | COG0168P | GO:0055085 | K03498: | potassium uptake protein KtrB |
| DR_1678 | 15.4899 | 2.53102 | -2.61353 | COG2153R | GO:0008080|GO:0016740 | | hypothetical protein |
| DR_1679 | 21.3629 | 7.26044 | -1.55698 | COG2227H | GO:0016740 |  | N-methyl-transferase-like protein |
| DR_1680 | 51.3932 | 20.515 | -1.3249 | COG0220R | GO:0036265 | K03439: | hypothetical protein |
| DR_1683 | 16.8726 | 2.62507 | -2.68426 | COG2808K | GO:0055114 | K07734: | transcriptional regulator |
| DR_1685 | 35.492 | 7.39489 | -2.26289 | COG1192D |  | K03496: | ParA family chromosome partitioning ATPase |
| DR_1686 | 48.6753 | 5.50817 | -3.14354 | - |  |  | hypothetical protein |
| DR_1687 | 38.9169 | 15.7038 | -1.30929 | COG0008J | GO:0046872 | K01885:ko00860 Porphyrin and chlorophyll metabolism | glutamyl-tRNA synthetase-like protein |
| DR_1696 | 19.5791 | 8.42414 | -1.21671 | COG0323L | GO:0030983 | K03572:ko03430 Mismatch repair | DNA mismatch repair protein MutL |
| DR_1710 | 175.934 | 63.069 | -1.48004 | - |  |  | hypothetical protein |
| DR_1750 | 35.0083 | 16.6762 | -1.0699 | - |  |  | hypothetical protein |
| DR_1807 | 52.5991 | 23.0241 | -1.19189 | COG2361S | GO:0016740 |  | hypothetical protein |
| DR_1821 | 19.6014 | 8.08134 | -1.27829 | - |  |  | hypothetical protein |
| DR_1855 | 7.84056 | 0 | -1.79769e+308 | COG1555L | GO:0006281 | K02237: | competence protein ComEA-like protein |
| DR_1864 | 33.148 | 15.0103 | -1.14297 | COG2363S |  |  | hypothetical protein |
| DR_1873 | 20.056 | 9.84148 | -1.02709 | - | GO:0004180 |  | hypothetical protein |
| DR_1877 | 8.52011 | 1.81034 | -2.23461 | COG1787V | GO:0090305 |  | hypothetical protein |
| DR_1881 | 23.7046 | 0 | -1.79769e+308 | - |  |  | hypothetical protein |
| DR_1884 | 35.5208 | 16.2012 | -1.13256 | COG1609K | GO:0006355 | K02529: | transcriptional regulator |
| DR_1886 | 149.457 | 46.8512 | -1.67358 | COG1999R |  | K07152: | hypothetical protein |
| DR_1887 | 122.993 | 45.6268 | -1.43062 | COG3336S |  | K02862: | hypothetical protein |
| DR_1888 | 57.2676 | 28.1545 | -1.02435 | - | GO:0016831 |  | hypothetical protein |
| DR_1901 | 40.1195 | 16.6376 | -1.26986 | - |  |  | hypothetical protein |
| DR_1903 | 68.6366 | 24.976 | -1.45844 | COG1307S |  |  | hypothetical protein |
| DR_1905 | 54.5508 | 26.8201 | -1.02429 | COG2186K | GO:0006355 |  | GntR family transcriptional regulator |
| DR_1906 | 117.733 | 34.0242 | -1.79089 | COG1620C | GO:0035873 | K03303: | L-lactate permease |
| DR_1910 | 52.1666 | 20.8146 | -1.32553 | COG3265G | GO:0016310|GO:0046316 | K00851:ko00030 Pentose phosphate pathway | thermoresistant gluconokinase |
| DR_1912 | 28.2692 | 8.2122 | -1.78339 | COG2365T | GO:0035335 |  | protein-tyrosine phosphatase |
| DR_1913 | 380.649 | 176.959 | -1.10504 | COG0188L | GO:0016853 | K02469: | DNA gyrase subunit A |
| DR_1914 | 20.7878 | 0 | -1.79769e+308 | - |  |  | hypothetical protein |
| DR_1930 | 83.0172 | 20.4741 | -2.01961 | - |  |  | hypothetical protein |
| DR_1931 | 155.548 | 7.34967 | -4.40354 | COG2267I |  | K06889: | hypothetical protein |
| DR_1934 | 45.6205 | 2.95914 | -3.94643 | - | GO:0016787 |  | hypothetical protein |
| DR_1935 | 120.167 | 42.7993 | -1.48938 | COG2390K | GO:0030246 |  | transcriptional regulator |
| DR_1965 | 11.4774 | 4.64683 | -1.30448 | COG2179R | GO:0016787 | K07015: | hypothetical protein |
| DR_1989 | 6.19595 | 2.53102 | -1.2916 | - |  |  | hypothetical protein |
| DR_1990 | 6.257 | 3.0898 | -1.01796 | - |  |  | hypothetical protein |
| DR_2012 | 36.6628 | 13.2551 | -1.46777 | - | GO:0016747 |  | hypothetical protein |
| DR_2037 | 4.4429 | 0 | -1.79769e+308 | - |  |  | hypothetical protein |
| DR_2052 | 69.9998 | 22.7636 | -1.62062 | COG1132V | GO:0055085 | K06147: | ABC transporter ATP-binding protein |
| DR_2081 | 217.785 | 95.335 | -1.19183 | COG0441J | GO:0046872 | K01868:ko00970 Aminoacyl-tRNA biosynthesis | threonyl-tRNA synthetase |
| DR_2082 | 70.8223 | 22.3906 | -1.66131 | - |  |  | hypothetical protein |
| DR_2095 | 285.437 | 109.234 | -1.38575 | COG2863C | GO:0020037 |  | c-type cytochrome |
| DR_2119 | 28.2422 | 10.0753 | -1.48703 | COG0411E | GO:0017111 | K01995:ko02010 ABC transporters | branched-chain amino acid ABC transporter ATP-binding protein |
| DR_2124 | 657688 | 328064 | -1.00343 | COG0257J | GO:0030529|GO:0009536 | K02919:ko03010 Ribosome | 50S ribosomal protein L36 |
| DR_2131 | 241.108 | 98.3673 | -1.29343 | - |  | K07028: | hypothetical protein |
| DR_2132 | 627.363 | 139.257 | -2.17155 | - | GO:0006950 |  | hypothetical protein |
| DR_2133 | 39.9873 | 17.0842 | -1.22687 | COG4372S |  |  | hypothetical protein |
| DR_2158 | 19.7769 | 6.88774 | -1.52171 | - |  |  | hypothetical protein |
| DR_2176 | 178.231 | 54.5355 | -1.70848 | COG1253R | GO:0050660 | K03699: | hypothetical protein |
| DR_2177 | 91.4736 | 35.0092 | -1.38562 | COG0295F | GO:0016787 | K01489:ko00240 Pyrimidine metabolism | cytidine deaminase |
| DR_2205 | 11.6999 | 3.68373 | -1.66726 | COG1289S |  |  | hypothetical protein |
| DR_2207 | 481.433 | 204.095 | -1.23809 | - |  |  | hypothetical protein |
| DR_2215 | 9.22106 | 3.69331 | -1.32002 | COG0561R |  |  | hypothetical protein |
| DR_2236 | 66.8399 | 17.5827 | -1.92655 | - |  |  | hypothetical protein |
| DR_2243 | 362.332 | 68.3912 | -2.40543 | COG0704P |  | K02039: | phosphate transport system regulatory protein PhoU |
| DR_2244 | 97.8009 | 3.22092 | -4.9243 | COG5002T | GO:0023014 |  | sensory transduction histidine kinase |
| DR_2245 | 319.841 | 49.2369 | -2.69954 | COG0745TK | GO:0035556 |  | phosphate regulon transcriptional regulatory protein PhoB |
| DR_2276 | 1356.37 | 0 | -1.79769e+308 | - | hypothetical protein |  |  |
| DR_2306 | 128.146 | 49.1625 | -1.38215 | COG0789K | GO:0006355 |  | MerR family transcriptional regulator |
| DR_2314 | 402.297 | 180.124 | -1.15927 | - |  |  | hypothetical protein |
| DR_2317 | 521.385 | 217.005 | -1.26462 | COG0667C | GO:0005216 |  | potassium channel subunit beta |
| DR_2322 | 291.513 | 32.8388 | -3.15009 | COG1404O | GO:0008233|GO:0043086 | | serine protease |
| DR_2325 | 345.188 | 24.554 | -3.81335 | COG1404O | GO:0043086 |  | serine protease |
| DR_2331 | 66.8667 | 31.7177 | -1.076 | COG0622R | GO:0016787 |  | hypothetical protein |
| DR_2337 | 5.80743 | 1.00984 | -2.52378 | - |  |  | hypothetical protein |
| DR_2368 | 19.7786 | 9.54584 | -1.051 | COG2860S |  |  | hypothetical protein |
| DR_2400 | 408.183 | 53.2646 | -2.93797 | COG2315S |  |  | hypothetical protein |
| DR_2401 | 270.18 | 46.5225 | -2.53792 | - |  |  | hypothetical protein |
| DR_2403 | 25.1144 | 5.84062 | -2.10432 | - |  |  | hypothetical protein |
| DR_2418 | 103.129 | 49.3987 | -1.06191 | COG0745TK | GO:0035556 |  | DNA-binding response regulator |
| DR_2454 | 49.5356 | 19.9141 | -1.31468 | COG1476K | GO:0043565 | K07729: | PbsX family transcriptional regulator |
| DR_2455 | 16.2472 | 2.65927 | -2.61109 | - |  |  | hypothetical protein |
| DR_2496 | 43.1066 | 19.0874 | -1.17529 | COG0771M | GO:0051301 | K01925:ko00471 D-Glutamine and D-glutamate metabolism | UDP-N-acetylmuramoylalanine--D-glutamiate ligase |
| DR_2529 | 363.391 | 140.049 | -1.3756 | - |  |  | hypothetical protein |
| DR_2546 | 21.2685 | 10.1918 | -1.06131 | COG4248R | GO:0016772 |  | hypothetical protein |
| DR_2547 | 226.844 | 107.529 | -1.07697 | COG0373H | GO:0055114 | K02492:ko00860 Porphyrin and chlorophyll metabolism | glutamyl-tRNA reductase |
| DR_2559 | 1107.94 | 491.457 | -1.17275 | - |  |  | hypothetical protein |
| DR_2581 | 41.2966 | 17.8218 | -1.21238 | COG1316K |  |  | LytR/CspA/Psr family protein |
| DR_2617 | 160.471 | 74.5182 | -1.10664 | COG1612O | GO:0055114 | K02259:ko00190 Oxidative phosphorylation | cytochrome AA3-controlling protein CtaA |
| DR_A0018 | 257.006 | 96.4318 | -1.41422 | COG0737F | GO:0046872 | K01081:ko00230 Purine metabolism | 5'-nucleotidase |
| DR_A0025 | 21.6465 | 9.62639 | -1.16906 | COG1804C | GO:0016740|GO:0033608|GO:0008152 | K07749: | hypothetical protein |
| DR_A0026 | 9.01439 | 3.18351 | -1.50161 | - |  |  | hypothetical protein |
| DR_A0030 | 32.5812 | 8.80463 | -1.88771 | COG1012C | GO:0055114 | K00294:ko00250 Alanine, aspartate and glutamate metabolism | 1-pyrroline-5-carboxylate dehydrogenase |
| DR_A0055 | 125.331 | 57.9863 | -1.11195 | COG0524G | GO:0016310|GO:0016740|GO:0016773 | K00852:ko00030 Pentose phosphate pathway | ribokinase |
| DR_A0077 | 9.5157 | 2.59356 | -1.87538 | - |  |  | hypothetical protein |
| DR_A0081 | 9.19675 | 3.82088 | -1.26722 | - |  |  | hypothetical protein |
| DR_A0093 | 2.93397 | 0.889967 | -1.72103 | - | GO:0045735 |  | hypothetical protein |
| DR_A0107 | 3.6308 | 0 | -1.79769e+308 | - |  |  | hypothetical protein |
| DR_A0113 | 11.114 | 1.98261 | -2.48691 | - |  |  | hypothetical protein |
| DR_A0114 | 6.73236 | 0 | -1.79769e+308 | - |  |  | hypothetical protein |
| DR_A0115 | 9.57072 | 0 | -1.79769e+308 | - |  |  | hypothetical protein |
| DR_A0119 | 21.1913 | 4.15251 | -2.35142 | - | GO:0032259 |  | hypothetical protein |
| DR_A0123 | 15.2769 | 6.17739 | -1.30628 | COG0394T | GO:0035335 | K03741: | arsenate reductase |
| DR_A0141 | 3985.03 | 1794.49 | -1.15101 | - |  |  | hypothetical protein |
| DR_A0142 | 116.506 | 55.3999 | -1.07245 | COG1309K | GO:0046677 |  | TetR family transcriptional regulator |
| DR_A0151 | 103.542 | 35.2741 | -1.55353 | COG2987E | GO:0016829 | K01712:ko00340 Histidine metabolism | urocanate hydratase |
| DR_A0156 | 28.4633 | 8.94405 | -1.6701 | COG1937S |  |  | hypothetical protein |
| DR_A0157 | 2063.17 | 3.99058 | -9.01405 | COG0226P | GO:0043190 | K02040:ko02010 ABC transporters | phosphate ABC transporter periplasmic phosphate-binding protein |
| DR_A0158 | 361.395 | 2.55175 | -7.14595 | COG0573P | GO:0016021 | K02037:ko02010 ABC transporters | phosphate ABC transporter permease |
| DR_A0159 | 227.994 | 1.77199 | -7.00748 | COG0581P | GO:0035435 | K02038:ko02010 ABC transporters | phosphate ABC transporter permease |
| DR_A0160 | 666.61 | 6.52827 | -6.674 | COG1117P | GO:0035435 | K02036:ko02010 ABC transporters | phosphate ABC transporter ATP-binding protein |
| DR_A0161 | 407.881 | 4.56533 | -6.48129 | COG0704P |  | K02039: | phosphate transport system regulatory protein PhoU |
| DR_A0163 | 17.936 | 0 | -1.79769e+308 | COG2391R |  | K07112: | hypothetical protein |
| DR_A0183 | 29.261 | 11.7224 | -1.31972 | COG2252R | GO:0055085 | K06901: | hypothetical protein |
| DR_A0209 | 115.28 | 43.6466 | -1.4012 | COG0601EP | GO:0016021 | K02033: | peptide ABC transporter permease |
| DR_A0211 | 12.315 | 0 | -1.79769e+308 | COG2188K | GO:0006355 | K03710: | GntR family transcriptional regulator |
| DR_A0218 | 24.802 | 10.3639 | -1.25889 | COG1680V |  |  | hypothetical protein |
| DR_A0243 | 136.208 | 54.0263 | -1.33408 | COG1017C | GO:0071949 | K05916:ko05132 Salmonella infection | flavohemoprotein |
| DR_A0244 | 68.0711 | 20.3544 | -1.7417 | COG1032C | GO:0051536 |  | hypothetical protein |
| DR_A0281 | 93.7676 | 36.374 | -1.36618 | - |  |  | hypothetical protein |
| DR_A0289 | 22.2085 | 2.87801 | -2.94797 | - |  |  | hypothetical protein |
| DR_A0312 | 96.7363 | 29.0145 | -1.73729 | COG0378OK | GO:0042803 | K03189: | urease accessory protein UreG |
| DR_A0313 | 17.7445 | 4.83574 | -1.87556 | COG0830O | GO:0016151 | K03188: | urease accessory protein UreF |
| DR_A0328 | 22.5746 | 8.77574 | -1.36311 | COG0654HC | GO:0055114 |  | oxidoreductase |
| DR_A0329 | 66.0038 | 26.4661 | -1.3184 | COG1819GC | GO:0016758|GO:0030259 | | glycosyltransferase |
| DR_A0339 | 15.1041 | 6.50392 | -1.21556 | COG3483E | GO:0055114 | K00453:ko00380 Tryptophan metabolism | tryptophan 2,3-dioxygenase |
| DR_A0348 | 91.1739 | 34.2221 | -1.41369 | COG1012C | GO:0055114 | K00128:ko00010 Glycolysis / Gluconeogenesis | aldehyde dehydrogenase |
| DR_B0005 | 21.7573 | 4.3416 | -2.3252 | - | GO:0006313 |  | transposase |
| DR_B0010 | 250.661 | 102.355 | -1.29216 | COG1270H | GO:0048472 | K02227:ko00860 Porphyrin and chlorophyll metabolism | cobalamin biosynthesis protein CobD |
| DR_B0011 | 228.252 | 72.035 | -1.66385 | COG0079E | GO:0030170 | K00817:ko00340 Histidine metabolism | histidinol-phosphate aminotransferase |
| DR_B0012 | 108.06 | 53.3421 | -1.01848 | COG1492H | GO:0015420|GO:0051188 | K02232:ko00860 Porphyrin and chlorophyll metabolism | cobyric acid synthase |
| DR_B0020 | 12.5269 | 5.92037 | -1.08127 | - | GO:0006313 |  | transposase |
| DR_B0025 | 96.306 | 22.321 | -2.10922 | COG1366T |  |  | sigma-B regulator RsbS |
| DR_B0026 | 84.3282 | 37.3183 | -1.17613 | COG2172T | GO:0005524|GO:0016310 | | sigma-B regulator RsbT |
| DR_B0046 | 275.475 | 9.76065 | -4.8188 |  |  |  |  |
| DR_B0047 | 52.1099 | 0 | -1.79769e+308 | - | GO:0016857|GO:0003676 | K01783:ko00030 Pentose phosphate pathway | hypothetical protein |
| DR_B0048 | 1411.23 | 10.3877 | -7.08593 | - |  |  | hypothetical protein |
| DR_B0049 | 218.695 | 7.5003 | -4.86583 | COG1136V | GO:0017111 | K02003: | ABC transporter, ATP-binding protein |
| DR_B0050 | 135.831 | 4.31015 | -4.97793 | - | GO:0016021 | K02004: | hypothetical protein |
| DR_B0051 | 70.8047 | 33.9198 | -1.06172 | - |  |  | hypothetical protein |
| DR_B0057 | 35.2731 | 14.9983 | -1.23377 | - | GO:0006313 |  | transposase |
| DR_B0058 | 5.05309 | 1.09641 | -2.20438 |  |  |  |  |
| DR_B0063 | 33.9502 | 15.5576 | -1.1258 | COG3188NU | GO:0016020 | K07347:ko05133 Pertussis | hypothetical protein |
| DR_B0064 | 78.2741 | 6.70804 | -3.54457 | - |  |  | hypothetical protein |
| DR_B0065 | 659.392 | 24.4997 | -4.7503 | - | GO:0005509|GO:0004527 | K07004: | hypothetical protein |
| DR_B0066 | 26.2131 | 8.13403 | -1.68824 | - |  |  | hypothetical protein |
| DR_B0067 | 1082.38 | 55.616 | -4.28257 | COG2374R | GO:0004527 | K07004: | extracellular nuclease |
| DR_B0072 | 30.717 | 8.08311 | -1.92605 | COG0654HC | GO:0004497|GO:0055114 | K14974:ko00760 Nicotinate and nicotinamide metabolism | salicylate monooxygenase-related protein |
| DR_B0074 | 36.5763 | 10.3336 | -1.82357 | COG1105G | GO:0016773 | K00882:ko00051 Fructose and mannose metabolism | 1-phosphofructokinase |
| DR_B0075 | 26.4365 | 7.70736 | -1.77822 |  |  |  |  |
| DR_B0077 | 48.4995 | 24.1489 | -1.00601 | COG3375S | GO:0008080 |  | hypothetical protein |
| DR_B0102 | 3.95587 | 1.57876 | -1.3252 | - | GO:0006313 |  | transposase |
| DR_B0115 | 20.277 | 3.89171 | -2.38137 | - |  |  | hypothetical protein |
| DR_B0133 | 14.1404 | 6.48011 | -1.12574 | COG1115E | GO:0016020 | K03310: | Na(+)-linked D-alanine glycine permease |
| DR_B0134 | 10.549 | 3.15753 | -1.74024 | - | GO:0006313 |  | transposase |
| DR_B0135 | 15.5418 | 7.52969 | -1.04549 | COG1205R | GO:0016787 |  | RNA helicase |
| DR_B0136 | 27.3031 | 12.7637 | -1.09702 | COG1061KL | GO:0005524 |  | ATP-dependent helicase HepA |
| DR_B0137 | 12.227 | 4.78907 | -1.35226 | - | GO:0032259 |  | hypothetical protein |
| DR_B0140 | 19.8239 | 5.25126 | -1.91651 | - | GO:0032259 |  | hypothetical protein |
| DR_C0001 | 96.8947 | 0 | -1.79769e+308 | COG2124Q | GO:0055114 |  | cytochrome P450-related protein |
| DR_C0002 | 258.39 | 0 | -1.79769e+308 | COG0574G | GO:0016772 | K01007:ko00620 Pyruvate metabolism | phosphoenolpyruvate synthase-related protein |
| DR_C0003 | 264.128 | 0 | -1.79769e+308 | COG0574G | GO:0016310|GO:0016772 | K01007:ko00620 Pyruvate metabolism | phosphoenolpyruvate synthase-related protein |
| DR_C0005 | 85.2523 | 0 | -1.79769e+308 | COG1961L | GO:0006310 |  | resolvase |
| DR_C0006 | 50.5522 | 0 | -1.79769e+308 | COG0084L | GO:0046872|GO:0016888 | K03424: | hypothetical protein |
| DR_C0007 | 142.344 | 0.290005 | -8.93908 | COG0603R |  |  | hypothetical protein |
| DR_C0008 | 69.953 | 0 | -1.79769e+308 | - |  |  | hypothetical protein |
| DR_C0009 | 85.1171 | 0 | -1.79769e+308 | COG4928R |  |  | hypothetical protein |
| DR_C0010 | 8.38547 | 0 | -1.79769e+308 | - | GO:0004519 |  | hypothetical protein |
| DR_C0011 | 34.2338 | 0 | -1.79769e+308 |  |  |  |  |
| DR_C0012 | 11.0024 | 0 | -1.79769e+308 | COG2197TK | GO:0043565 |  | GerE family transcriptional regulator |
| DR_C0013 | 28.8063 | 0 | -1.79769e+308 | COG0860M | GO:0009253|GO:0016787 | K01448: | N-acetylmuramoyl-L-alanine amidase, putative |
| DR_C0014 | 246.758 | 0 | -1.79769e+308 | - |  |  | hypothetical protein |
| DR_C0015 | 171.845 | 0 | -1.79769e+308 | COG1520S |  |  | hypothetical protein |
| DR_C0016 | 18.8265 | 0 | -1.79769e+308 | - |  |  | hypothetical protein |
| DR_C0017 | 24.6343 | 0 | -1.79769e+308 | COG4644L | GO:0006313 |  | putative transposase |
| DR_C0018 | 37.3773 | 0 | -1.79769e+308 | COG4974L | GO:0015074 |  | integrase/recombinase XerD, putative |
| DR_C0020 | 146.796 | 0 | -1.79769e+308 | COG0863L | GO:0090124|GO:0008170 | | modification methylase, putative |
| DR_C0021 | 30.5508 | 0 | -1.79769e+308 | - |  |  | hypothetical protein |
| DR_C0022 | 93.8046 | 0 | -1.79769e+308 | - |  |  | hypothetical protein |
| DR_C0023 | 138.864 | 0 | -1.79769e+308 | - |  |  | hypothetical protein |
| DR_C0024 | 112.943 | 0 | -1.79769e+308 | - |  |  | hypothetical protein |
| DR_C0025 | 165.51 | 0 | -1.79769e+308 | COG1192D |  | K03496: | ParA family chromosome partitioning ATPase |
| DR_C0026 | 109.532 | 0 | -1.79769e+308 | - |  |  | hypothetical protein |
| DR_C0027 | 145.323 | 0 | -1.79769e+308 | - |  |  | hypothetical protein |
| DR_C0028 | 122.212 | 0 | -1.79769e+308 | COG3436L |  | K07484: | transposase, putative |
| DR_C0030 | 74.1995 | 0.282969 | -8.03462 | - |  |  | hypothetical protein |
| DR_C0032 | 72.6951 | 0 | -1.79769e+308 | - | GO:0006313 |  | putative transposase |
| DR_C0034 | 103.626 | 0 | -1.79769e+308 | COG0535R | GO:0051539|GO:0051536 | | coenzyme PQQ synthesis protein, putative |
| DR_C0035 | 206.935 | 0 | -1.79769e+308 | - |  |  | hypothetical protein |
| DR_C0036 | 207.571 | 0.465537 | -8.80049 | COG1032C | GO:0051536 |  | oxidative cyclase, putative |
| DR_C0037 | 118.738 | 0.327024 | -8.50417 | COG2192O | GO:0009058|GO:0016740 | K00612: | nodulation protein-related protein |
| DR_C0038 | 277.275 | 0 | -1.79769e+308 | COG0312R |  | K03568: | hypothetical protein |
| DR_C0039 | 145.019 | 0 | -1.79769e+308 | - |  |  | hypothetical protein |
| DR_C0040 | 121.14 | 0 | -1.79769e+308 | - | GO:0055085 |  | multidrug-efflux transporter, putative |
| DR_C0041 | 108.367 | 0 | -1.79769e+308 | - |  |  | hypothetical protein |
| DR_r04 | 31345.9 | 0 | -1.79769e+308 |  |  |  |  |
| DR_t05 | 169575 | 0 | -1.79769e+308 |  |  |  |  |

Table S3: Real-time PCR result, Negative value means repression and positive value means induction.

| Gene | *dr2523* | *dr2283* | *dr1709* | *dr1236* | *dr1998* | *dr1506* | *dr0348* | *dr0828* |
| --- | --- | --- | --- | --- | --- | --- | --- | --- |
| **Normal (M/R1)：** |  |  |  |  |  |  |  |  |
| Mean (fold change) | 1.96 | 4.24 | 4.41 | -24.32 | -2.69 | -20.54 | -4.17 | 3.39 |
| SD | 0.71 | 1.41 | 2.82 | 19.79 | 5.26 | 19.57 | 3.82 | 1.83 |
| **Mn stress**： (M-Mn/R1-Mn) |  |  |  |  |  |  |  |  |
| Mean (fold change) | 20.14 | 2.25 | 1.86 | -53.94 | 1.91 | -1.53 | -1.44 | 1.76 |
| SD | 4.04 | 0.15 | 0.28 | 26.64 | 1.16 | 0.24 | 0.09 | 0.58 |

Figure S1: CLUSTAL W software was used to align amino acid sequences of *D. radiodurans*, *E. coli*, *P. aeroginosae*, *A. ferrooxidans*, *B. subtilis*, *M. marinum* and *H. pylori*. Amino acids from domain II, III, and I are highlighted in red, green and blue, respectively.

**Figure S2:** Schematic representation of homologous recombination used in *dr-0865* deletion.

**Figure S3:** Manganese sensitivity assay for wild–type R1, Mt-0865, and C-0865. Photos show the inhibition zone of wild-type R1 (A), Mt-0865 (B) and C-0865 (C). Histogram showing the results of Manganese sensitivity disk assay. Wild type R1, Mt-0865 and C-0865 were culture in TGY plates overlaid with filter discs saturated with 1M solution of manganese chloride and manganese sulfate.

**Figure S4:** The inhibition zone of the wild-type R1 strain (A), Mt-0865 strain (B) and C-0865 strain (C).
